# Whole-Body Hypothermia vs Targeted Normothermia for Neonates With Mild Encephalopathy: A Multicenter Pilot Randomized Clinical Trial

**DOI:** 10.1001/jamanetworkopen.2024.9119

**Published:** 2024-05-06

**Authors:** Paolo Montaldo, Mario Cirillo, Constance Burgod, Elisabetta Caredda, Serena Ascione, Mauro Carpentieri, Simona Puzone, Alessandra D’Amico, Reema Garegrat, Marianna Lanza, Maria Moreno Morales, Gaurav Atreja, Vijaykumar Shivamurthappa, Ujwal Kariholu, Narendra Aladangady, Paul Fleming, Asha Mathews, Balamurugan Palanisami, Joanne Windrow, Karen Harvey, Aung Soe, Santosh Pattnayak, Palaniappan Sashikumar, Sundeep Harigopal, Ronit Pressler, Martin Wilson, Enrico De Vita, Seetha Shankaran, Sudhin Thayyil

**Affiliations:** 1 Centre for Perinatal Neuroscience, Department of Brain Sciences, Imperial College London, London, United Kingdom; 2 Department of Woman, Child, and General and Specialized Surgery, University of Campania “Luigi Vanvitelli,” Naples, Italy; 3 Department of Advanced Medical and Surgical Sciences, MRI Research Center, University of Campania “Luigi Vanvitelli,” Naples, Italy; 4 Department of Radiology, “Tortorella” Private Hospital, Salerno, Italy; 5 Neonatal Unit, Imperial Health Care NHS Trust, London, United Kingdom; 6 Neonatal Unit, Homerton Healthcare NHS Foundation Trust, London, United Kingdom; 7 Centre for Paediatrics, Barts and the London School of Medicine and Dentistry, Queen Mary University of London, London, United Kingdom; 8 Liverpool Women’s NHS Foundation Trust, Liverpool, United Kingdom; 9 Oliver Fisher Neonatal Intensive Care Unit, Medway Maritime Hospital, Medway NHS Foundation Trust, Kent, United Kingdom; 10 Neonatal Medicine, Royal Victoria Infirmary, Newcastle Upon Tyne, United Kingdom; 11 Department of Neurophysiology, Great Ormond Street Hospital, London, United Kingdom; 12 Centre for Human Brain Health and School of Psychology, University of Birmingham, Birmingham, United Kingdom; 13 MRI Physics, Radiology Department, Great Ormond Street Hospital for Children NHS Foundation Trust, London, United Kingdom; 14 Department of Neonatal-Perinatal Medicine, Wayne State University, Detroit, Michigan; 15 Department of Pediatrics, The University of Texas at Austin, Dell Children’s Hospital, Austin, Texas

## Abstract

**Importance:**

Although whole-body hypothermia is widely used after mild neonatal hypoxic-ischemic encephalopathy (HIE), safety and efficacy have not been evaluated in randomized clinical trials (RCTs), to our knowledge.

**Objective:**

To examine the effect of 48 and 72 hours of whole-body hypothermia after mild HIE on cerebral magnetic resonance (MR) biomarkers.

**Design, Setting, and Participants:**

This open-label, 3-arm RCT was conducted between October 31, 2019, and April 28, 2023, with masked outcome analysis. Participants were neonates at 6 tertiary neonatal intensive care units in the UK and Italy born at or after 36 weeks’ gestation with severe birth acidosis, requiring continued resuscitation, or with an Apgar score less than 6 at 10 minutes after birth and with evidence of mild HIE on modified Sarnat staging. Statistical analysis was per intention to treat.

**Interventions:**

Random allocation to 1 of 3 groups (1:1:1) based on age: neonates younger than 6 hours were randomized to normothermia or 72-hour hypothermia (33.5 °C), and those 6 hours or older and already receiving whole-body hypothermia were randomized to rewarming after 48 or 72 hours of hypothermia.

**Main Outcomes and Measures:**

Thalamic *N*-acetyl aspartate (NAA) concentration (mmol/kg wet weight), assessed by cerebral MR imaging and thalamic spectroscopy between 4 and 7 days after birth using harmonized sequences.

**Results:**

Of 225 eligible neonates, 101 were recruited (54 males [53.5%]); 48 (47.5%) were younger than 6 hours and 53 (52.5%) were 6 hours or older at randomization. Mean (SD) gestational age and birth weight were 39.5 (1.1) weeks and 3378 (380) grams in the normothermia group (n = 34), 38.7 (0.5) weeks and 3017 (338) grams in the 48-hour hypothermia group (n = 31), and 39.0 (1.1) weeks and 3293 (252) grams in the 72-hour hypothermia group (n = 36). More neonates in the 48-hour (14 of 31 [45.2%]) and 72-hour (13 of 36 [36.1%]) groups required intubation at birth than in the normothermic group (3 of 34 [8.8%]). Ninety-nine neonates (98.0%) had MR imaging data and 87 (86.1%), NAA data. Injury scores on conventional MR biomarkers were similar across groups. The mean (SD) NAA level in the normothermia group was 10.98 (0.92) mmol/kg wet weight vs 8.36 (1.23) mmol/kg wet weight (mean difference [MD], −2.62 [95% CI, −3.34 to −1.89] mmol/kg wet weight) in the 48-hour and 9.02 (1.79) mmol/kg wet weight (MD, −1.96 [95% CI, −2.66 to −1.26] mmol/kg wet weight) in the 72-hour hypothermia group. Seizures occurred beyond 6 hours after birth in 4 neonates: 1 (2.9%) in the normothermia group, 1 (3.2%) in the 48-hour hypothermia group, and 2 (5.6%) in the 72-hour hypothermia group.

**Conclusions and Relevance:**

In this pilot RCT, whole-body hypothermia did not improve cerebral MR biomarkers after mild HIE, although neonates in the hypothermia groups were sicker at baseline. Safety and efficacy of whole-body hypothermia should be evaluated in RCTs.

**Trial Registration:**

ClinicalTrials.gov Identifier: NCT03409770

## Key Points

**Question:**

Does whole-body hypothermia initiated within 6 hours of birth and continued for 48 or 72 hours increase thalamic magnetic resonance (MR) spectroscopy *N*-acetyl aspartate levels in neonates with mild hypoxic-ischemic encephalopathy (HIE) compared with normothermia?

**Findings:**

In this pilot randomized clinical trial (RCT) of 101 neonates with mild HIE, whole-body hypothermia initiated within 6 hours after birth and continued for 48 or 72 hours did not improve cerebral MR biomarkers, although neonates in the hypothermic groups were more unwell at baseline.

**Meaning:**

Carefully designed RCTs to evaluate the impact of whole-body hypothermia on neurodevelopmental outcomes after mild HIE are urgently required.

## Teaser

This pilot randomized clinical trial examines the effect of 48 and 72 hours of whole-body hypothermia compared with normothermia on cerebral magnetic resonance biomarkers among neonates with mild hypoxic-ischemic encephalopathy.

## Introduction

Whole-body hypothermia is the standard treatment for neonates with moderate or severe hypoxic-ischemic encephalopathy (HIE) in high-income countries.^1,2,3,4^ Although to our knowledge its safety and efficacy for mild HIE have not been evaluated in randomized clinical trials (RCTs), the Canadian Neonatal Network,^5^ California Perinatal Transport System,^6^ and New South Wales in Australia^7^ have reported that greater than 80% of neonates with mild HIE receive whole-body hypothermia. In the UK, many cooled neonates do not even have encephalopathy,^8^ and neurological assessments are rarely performed before this treatment.^8,9^ This widespread therapeutic drift has raised concerns about lack of equipoise for conducting an RCT of whole-body hypothermia for mild HIE.^10^

Thalamic proton magnetic resonance (MR) spectroscopy biomarkers predict adverse neurodevelopmental outcomes at 18 months after moderate or severe encephalopathy.^11,12^ In mild HIE, brain injury is primarily seen in white matter rather than deep brain nuclei on conventional MR imaging (MRI). However, white matter is associated with metabolic perturbations in the thalamus, even in the absence of visible injury to deep brain nuclei on conventional MRI.^13^ Although standard duration of whole-body hypothermia for moderate and severe HIE is 72 hours, in a murine model of neonatal mild hypoxia-ischemia even a short period (3.5 hours) of hypothermia was highly neuroprotective.^14^ We examined the effects of whole-body hypothermia, initiated within 6 hours of birth and continued for either 72 or 48 hours, on MRI and spectroscopy biomarkers compared with normothermia among neonates with mild HIE.

## Methods

### Study Design and Participants

**Figure 1.**
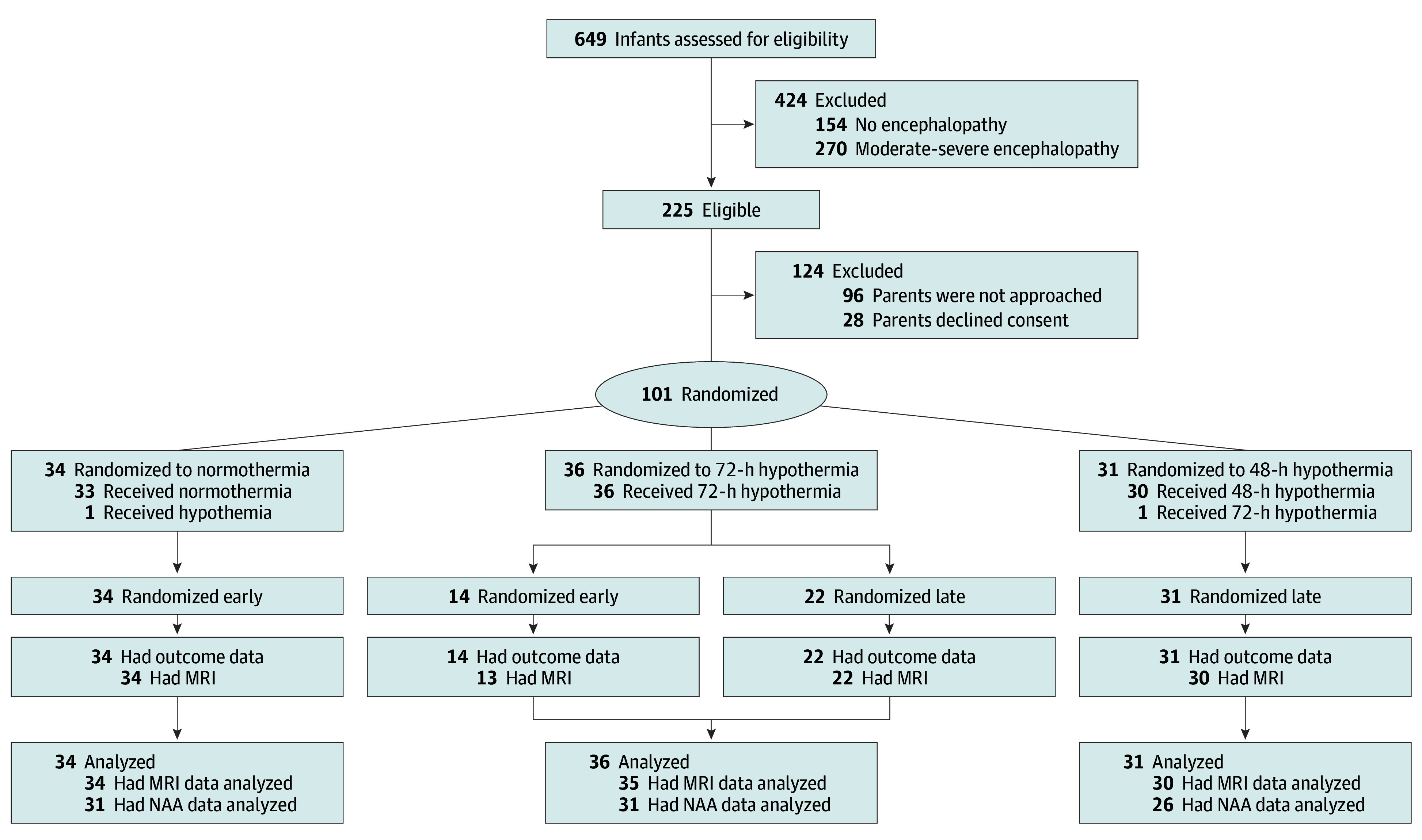
Flowchart Early randomization occurred within 6 hours and late randomization, when the neonate was 6 hours or older. MRI indicates magnetic resonance imaging; NAA, *N*-acetyl aspartate.

This open-label, multicountry pilot RCT recruited neonates with mild HIE from 6 tertiary neonatal intensive care units (NICUs) in the UK and Italy between October 31, 2019, and April 28, 2023. The clinical trial protocol (Supplement 1) was approved by the UK national and University of Campania “Luigi Vanvitelli” ethics committees and was sponsored by Imperial College London. Written informed parental consent was obtained before recruitment. We followed the Consolidated Standards of Reporting Trials (CONSORT) reporting guideline^15^ (Figure 1).

All neonates born at 36 weeks’ gestation or later with a birth weight of 2 kg or more requiring resuscitation at birth and admission to a NICU were screened for eligibility. Neonates were recruited to the trial if they met 3 criteria. The first was evidence of recent intrapartum hypoxia-ischemia (10-minute Apgar score <6; continued need for resuscitation, including endotracheal or mask ventilation, at 10 minutes after birth; and/or birth acidosis, defined as pH of <7.0 or base excess of >16 mmol/L in any cord or neonate gas sample within 60 minutes of birth). If the pH or base excess was borderline (<7.15 to 7.0 and/or 10-16 mmol/L, respectively) in the umbilical cord and/or any blood gas within 1 hour of birth or no blood gas was available, additional evidence of perinatal asphyxia was required, including an acute obstetric event (eg, late or variable decelerations, cord prolapse, cord rupture, uterine rupture, maternal trauma, hemorrhage, or acute cardiorespiratory arrest). The second criterion was evidence of mild HIE, defined as 2 or more abnormal findings in any of the 6 modified Sarnat examination categories (consciousness level, spontaneous activity, posture, tone, primitive reflexes, and autonomic nervous system) but not meeting the diagnosis of moderate or severe HIE (≥3 moderate or severe abnormalities) on a standardized examination performed by a certified examiner between 1 and 6 hours of age.^1,16^ Based on neurological examination at recruitment, we calculated a total Sarnat score for each neonate by adding weighted scores (0, normal; 1, mild; 2, moderate; or 3, severe) assigned for each of the 6 categories, as previously described.^17^ The examination was repeated daily until day 3 after birth. The third criterion was normal amplitude on amplitude-integrated electroencephalography (aEEG) performed for at least 30 minutes between 1 and 6 hours of age. Normal amplitude was defined as an upper margin of aEEG activity greater than 10 μV and lower margin greater than 5 μV on a single-channel aEEG. Neonates without encephalopathy or with moderate or severe HIE, who had seizures or had received antiseizure medications before randomization, or who had moderate or severe abnormalities on aEEG voltage^18^ or life-threatening congenital malformations were excluded.

Conventional MRI was performed between 4 and 7 days after birth on a 3-T scanner (Philips or GE HealthCare) with harmonized protocols, including standard 3-dimensional T1-weighted and 2-dimensional T2-weighted sequences and diffusion tensor imaging, proton MR spectroscopy metabolite peak area ratios, and metabolite absolute concentrations.^12^ The MR spectroscopy was acquired in a single 15 × 15 × 15-mm^3^ voxel centered on the left thalamus.

The MR scans were reported centrally by 2 neonatal neurologists (P.M., S.T.) with more than 10 years’ experience with MRI using a validated scoring system^19^; they were masked to the allocation. We summated regional scores from the cortex, basal ganglia and thalami, white matter, and posterior limb of the internal capsule to obtain a total MR injury score as previously described.^20^ Pseudonymized raw MR data were analyzed centrally after quality check to exclude poor-quality data but before analysis and were masked to the allocation. The MR spectroscopy data were analyzed with the ABfit algorithm^21^ implemented in the spant package, version 2.14.0^22^ of R, version 4.3.1 (R Project for Statistical Computing).^23^ The ABfit algorithm incorporates a spline basis into a frequency-domain analysis model with a penalty parameter to enforce baseline smoothness, thus performing accurate baseline estimation.^21^

### Randomization

Neonates were randomized to 1 of the 3 groups (1:1:1) based on age at randomization using a web-based randomization program (Sealed Envelope). The minimization method^24^ was used to control for severity, with a probability of 0.85 within each cohort for assigning to the treatment group that minimized imbalance. Severity of HIE was calculated from the number of categories under normal, mild, moderate, or severe from the modified Sarnat neurological examination at randomization: (1) at least 2 categories were mild and none were moderate or severe or (2) 1 or 2 categories were moderate or severe and all others were mild or normal. Neonates with fewer than 2 abnormalities or 3 or more moderate or severe categories were ineligible (eFigure 2 in Supplement 2).

### Intervention

Neonates recruited within 6 hours after birth and before initiation of hypothermia were randomized to either normothermia or therapeutic hypothermia for 72 hours at 33.5 °C (early randomization group). Neonates with mild HIE who were already receiving therapeutic hypothermia at 33.5 °C and met eligibility criteria were randomized to rewarming at either 48 or 72 hours of age (late randomization group).

Neonates randomized to either the 72- or 48-hour hypothermia arms were placed on a blanket attached to the cooling system. A rectal probe was inserted, and the core temperature was lowered to 33.5 °C by using the blanket’s servomechanism in both study groups, followed by automated rewarming at 0.5 °C per hour. No additional heat source was used during cooling.

In the 48- and 72-hour hypothermia groups, rectal and skin temperatures were monitored continuously and recorded hourly for the first 4 hours and every 2 hours during the remaining cooling period. Neonates undergoing hypothermia were sedated if distressed (shivering and/or unexplained tachycardia). In the normothermia group, only skin temperatures were monitored and recorded hourly for the first 4 hours and every 2 hours until 80 hours after randomization. Temperature was maintained within the target range (36.5-37.5 °C) using a radiant warmer or servo-controlled incubator during the initial period, followed by clothing and blankets without an external heat source.

### Outcomes

The primary outcome was thalamic *N*-acetyl aspartate (NAA) concentration (mmol/kg wet weight). Secondary outcomes included ratios of thalamic lactate to NAA and NAA to creatine peak area metabolite, brain injury scores on conventional MRI at age 1 week, and mean duration of hospital stay.

### Adverse Events

Adverse events included cardiac arrhythmia, persistent acidosis, alteration of skin integrity, abnormal blood clotting, and thrombocytopenia requiring platelets. Serious adverse events included cardiac arrhythmia, life-threatening bleeds, or major venous thrombosis not related to an infusion line.

### Statistical Analysis

As this was a pilot RCT, no formal sample size calculations were performed. Clinical data were compared between the normothermia and 48-hour hypothermia groups and the normothermia and 72-hour hypothermia groups using the Mann-Whitney test for continuous variables and the χ^2^ or Fisher exact test for categorical variables. Differences in outcomes were quantified as risk ratios (RRs) with 95% CIs. Ordinal logistic regression was used to analyze ordinal outcomes (MR conventional biomarkers), with group differences expressed as odds ratios with 95% CIs. Linear regression was used to analyze continuous outcomes (MR spectroscopy biomarkers), with group differences expressed as mean or median differences with 95% CIs. Analyses were performed per intention to treat with IBM SPSS, version 24 (IBM Corp). Two-sided *P* < .05 was significant.

## Results

### Study Population

Of 225 eligible neonates with mild HIE, parents of 129 (57.3%) were approached for participation. Of these, parents of 28 declined and 101 neonates were recruited (47 females [46.5%], 54 males [53.5%]). Mean (SD) gestational age and birth weight were 39.5 (1.1) weeks and 3378 (380) grams in the normothermia group, 38.7 (0.5) weeks and 3017 (338) grams in the 48-hour hypothermia group, and 39.0 (1.1) weeks and 3293 (252) grams in the 72-hour hypothermia group (Table 1).

**Table 1.** Baseline Characteristics

Characteristic	Neonates^a^		
	Normothermia (n = 34)	Hypothermia for 48 h (n = 31)	Hypothermia for 72 h (n = 36)
Antenatal history			
Maternal age, mean (SD), y	30.9 (5.9)	32.1 (5.3)	31.5 (6.2)
Primigravida	16 (47.1)	18 (58.1)	15 (41.7)
Reduced fetal movements, No./total No. (%)	1/25 (4.0)	3/24 (12.5)	1/27 (3.7)
CTG abnormalities, No./total No. (%)	16/21 (76.2)	10/28 (35.7)	16/30 (53.3)
Meconium staining, No./total No. (%)	15/34 (44.1)	8/31 (25.8)	8/34 (23.5)
Maternal pyrexia, No./total No. (%)	4/33 (12.1)	4/30 (13.3)	2/36 (5.6)
Prolonged rupture of membranes	2 (5.9)	2 (6.5)	3 (8.3)
Perinatal sentinel events, No./total No. (%)^b^	22/33 (66.7)	16/30 (53.3)	22/36 (61.1)
Cord mishap	14 (42.4)	3 (10.0)	9 (25.0)
Lengthened second stage	3 (9.1)	2 (6.7)	1 (2.8)
Obstructed labor and/or difficult extraction	2 (6.1)	4 (13.3)	5 (13.9)
Shoulder dystocia	4 (12.1)	3 (10.0)	2 (5.6)
Antepartum hemorrhage	1 (3.0)	3 (10.0)	4 (11.1)
Uterine rupture	0	1 (3.3)	2 (5.6)
Delivery			
Instrumental delivery	6 (17.6)	6 (19.4)	8 (22.2)
Emergency cesarean	9 (26.5)	13 (41.9)	16 (44.4)
Birth			
Inborn	28 (82.4)	13 (41.9)	23 (63.9)
Sex			
Female	15 (44.1)	13 (41.9)	19 (52.8)
Male	19 (55.9)	18 (58.1)	17 (47.2)
Gestational age, mean (SD), wk	39.5 (1.1)	38.7 (0.5)	39.0 (1.1)
Birth weight, mean (SD), g	3378 (380)	3017 (338)	3293 (252)
Cord arterial blood pH, mean (SD)	7.0 (0.1)	7.0 (0.2)	7.0 (0.1)
Apgar score, median (IQR)			
5 min	7.0 (6.0-8.0)	5.0 (3.0-7.0)	6.0 (4.0-7.0)
10 min	8.0 (7.0-9.0)	7.0 (6.0-9.0)	8.0 (6.0-9.0)
Delivery room resuscitation			
Intubation	3 (8.8)	14 (45.2)	13 (36.1)
Epinephrine	0	1 (3.2)	0
Chest compression	0	5 (16.1)	0
Extubation within 3 h	1 (2.9)	5 (16.1)	2 (5.6)

Abbreviation: CTG, cardiotocography.

^a^ Data are presented as the number (percentage) of neonates unless otherwise indicated.

^b^ Events were not mutually exclusive.

Of the 101 neonates, 48 (47.5%) were younger than 6 hours and allocated to normothermia (34 [70.8%]) or 72 hours of whole-body hypothermia (14 [29.2%]). Fifty-three neonates (52.5%) who were 6 hours or older who had whole-body hypothermia initiated within 6 hours as clinical care were allocated to rewarming at 48 hours (31 [58.5%]) or 72 hours (22 [41.5%]). Among those randomized within 6 hours, 41 (85.4%) were inborn and 7 (14.6%), outborn; among those randomized when 6 hours or older, 23 (43.4%) were inborn and 30 (56.6%), outborn.

Median (range) ages at admission to the cooling center were 1.0 (0.3-1.4), 3.3 (0.4-7.0), and 0.7 (0.3-5.2) hours and at randomization were 4.0 (1.1-5.8), 36.3 (6.1-47.3), and 28.8 (1.3-47.1) hours in the normothermia, 48-hour hypothermia, and 72-hour hypothermia groups, respectively. Combined data for the 3 randomization groups are presented in Table 1 and Table 2, and separate data on the 4 subgroups (normothermia, 48-hour hypothermia, and 72-hour hypothermia randomized within 6 hours and when 6 hours or older) and recruitment per site are in eTable 1 and eFigure 1 in Supplement 2.

**Table 2.** Short-Term Outcomes During Neonatal Hospitalization

Short-term outcome	Neonates^a^			RR or MD (95% CI)^b^	
	Normothermia (n = 34)	48-h Hypothermia (n = 31)	72-h Hypothermia (n = 36)	48-h Hypothermia vs normothermia	72-h Hypothermia vs normothermia
Invasive ventilation	3 (8.8)	14 (45.2)	16 (44.4)	5.11 (1.62 to 16.13)	5.03 (1.61 to 15.75)
Duration of invasive ventilation, median (IQR), h^c^	3 (2 to 3)	7 (1 to 16)	21 (9 to 41)	4 (−2.0 to 8.0)	18 (0.0 to 24.0)
Noninvasive ventilation^c^	8 (23.5)	1 (3.2)	5 (13.8)	0.13 (0.01 to 1.03)	0.59 (0.21 to 1.62)
Duration of noninvasive ventilation, median (IQR), h	3 (2 to 3)	18 (7 to 29)	59 (36 to 76)	15 (4.0 to 16.4)^d^	56 (3.0 to 71.0)^d^
Opioid use	0	26 (83.9)	29 (80.6)	NA	NA
Shivering	0	13 (41.9)	21 (58.3)	NA	NA
Hypotension requiring inotropes	1 (2.9)	0	4 (11.1)	NA	3.77 (0.44-32.12)
Persistent metabolic acidosis	0	0	1 (2.8)	0	NA
Subcutaneous fat necrosis	0	1 (3.2)	0	NA	0
Thrombocytopenia requiring platelets	1 (2.9)	1 (3.2)	2 (5.6)	1.09 (0.07 to 16.79)	1.88 (0.17 to 19.89)
Abnormal clotting	1 (2.9)	0	4 (11.1)	NA	3.77 (0.44 to 32.12)
Bloodstream infection	1 (2.9)	1 (3.2)	1 (2.8)	1.09 (0.07 to 16.79)	0.94 (0.06 to 14.50)
Seizures after 6 h of age	1 (2.9)	1 (3.2)	2 (5.6)	1.09 (0.07 to 16.79)	1.88 (0.17 to 19.89)
Duration of hospital stay, median (IQR), d	5.9 (3.7 to 6.6)	6.2 (4.8 to 7.9)	7.8 (6.2 to 9.1)	0.30 (−1.24 to 1.73)^d^	1.91 (0.60 to 3.59)^d^
Death	0	0	1 (2.8)	0	NA

Abbreviations: MD, median difference; NA, not applicable; RR, risk ratio.

^a^ Data are presented as the number (percentage) of neonates unless otherwise indicated.

^b^ Risk ratios were calculated as the risk of being in the next-highest outcome category for the hypothermia group relative to the risk of being in next-highest outcome category for the normothermia group.

^c^ Defined as the need for continuous positive airway pressure or high-flow oxygen.

^d^ Denotes MD (95% CI).

Temperature profiles are provided in eFigure 3 in Supplement 2. No neonate had hyperthermia greater than 38 °C during the first 3 days after birth. One (2.9%) in the normothermia and 1 (3.2%) in the 48-hour hypothermia group had temperatures between 37.5 and 38 °C.

Short-term outcomes are in Table 2. More neonates randomized to 48-hour and 72-hour hypothermia required invasive ventilation (14 [45.2%]; RR, 5.1 [95% CI, 1.6-16.1] and 16 [44.4%]; RR, 5.0 [95% CI, 1.6-15.8], respectively) than the normothermic group (3 [8.8%]). Median duration of invasive ventilation was longer than the normothermic group by 4 (95% CI, −2.0 to 8.0) hours in the 48-hour hypothermia group and by 18 (95% CI, 0.0-24.0) hours in the 72-hour group. Although no neonates randomized within 6 hours were sedated at neurological assessment, opioid administration during the first 2 days occurred in 26 of 31 (83.9%) in the 48-hour and 29 of 36 (80.6%) in the 72-hour group. Shivering during the intervention was reported in 13 of 31 (41.9%) in the 48-hour and 21 of 36 (58.3%) in the 72-hour group but none in the normothermia group.

The median (IQR) hospital stay was longer in the 72-hour hypothermia group (7.8 [6.2-9.1] days) than the normothermia group (5.9 [3.7-6.6] days) (median difference, 1.91 [95% CI, 0.60-3.59] days) (Table 2). In the 72-hour group, 1 neonate (2.8%) died at 24 hours after birth due to early-onset *Escherichia coli* sepsis with subsequent septic shock. No other serious adverse events were reported.

Four recruited neonates (4.0%) developed electroclinical seizures beyond 6 hours after birth: 1 each in the normothermia (2.9%) and 48-hour (3.2%) groups and 2 (5.6%) in the 72-hour group. The neonate in the normothermia group was started on whole-body hypothermia at 23 hours of age when seizures were noted on aEEG. However, at 48 hours of age, this neonate developed refractory hypotension and persistent pulmonary hypertension requiring inhaled nitric oxide, and whole-body hypothermia was discontinued. The MRI brain scan showed a cerebral abscess, and the blood culture was positive for *Enterobacter cloacae*. One neonate (3.2%) in the 48-hour hypothermia group developed electroclinical seizures at 33 hours after birth; hence, whole-body hypothermia was continued for 72 hours. Both neonates were analyzed as per intention to treat.

### MR Biomarkers

Magnetic resonance imaging, performed in 99 neonates (98.0%), showed normal results in 18 of 34 (52.9%) in the normothermia, 8 of 30 (26.7%) in the 48-hour, and 12 of 35 (34.3%) in the 72-hour groups. Median (IQR) age at MRI was 5.8 (5.0-7.0) days in the normothermia, 4.0 (3.5-5.8) days in the 48-hour, and 7.0 (5.7-8.4) days in the 72-hour groups. Brain injury was primarily in the white matter and partly in the cortical region. Only 1 neonate (1.0%) in the 72-hour group had injury to the basal ganglia or thalami (Table 3). The median (IQR) MRI total injury score was 0 (0-2) in the normothermia, 1 (0-2) in the 48-hour, and 1 (0-1) in the 72-hour groups.

**Table 3.** Conventional MRI and Spectroscopy

MR biomarker	Neonates^a^			OR or MD (95% CI)^b^	
	Normothermia (n = 34)	48-h Hypothermia (n = 30)	72-h Hypothermia (n = 35)	48-h Hypothermia vs normothermia	72-h Hypothermia vs normothermia
Postnatal age at MRI, median (IQR), d	5.8 (5.0 to 7.0)	4.0 (3.5 to 5.8)	7.0 (5.7 to 8.4)	−1.80 (−3.40 to 0.15)	1.20 (−0.52 to 3.00)
Basal ganglia and thalami injuries, No.					
0	34 (100)	30 (100)	34 (97.1)	NA	NA
1	0	0	0		
2	0	0	1 (2.9)		
3	0	0	0		
Posterior limb of internal capsule					
Normal	34 (100)	30 (100)	35 (100)	NA	NA
Equivocal	0	0	0		
Abnormal	0	0	0		
White matter injuries, No.					
0	18 (52.9)	8 (26.7)	12 (34.3)	2.10 (0.83 to 5.34)	1.73 (0.70 to 4.29)
1	9 (26.5)	16 (53.3)	16 (45.7)		
2	7 (20.6)	6 (20.0)	7 (20.0)		
3	0	0	0		
Cortex injuries, No.					
0	27 (79.4)	27 (90.0)	32 (91.4)	0.42 (0.10 to 1.78)	0.35 (0.08 to 1.50)
1	6 (17.6)	3 (10.0)	3 (8.6)		
2	1 (2.9)	0	0		
3	0	0	0		
Total injury score					
0	18 (52.9)	8 (26.7)	12 (34.3)	1.64 (0.66 to 4.11)	1.41 (0.57 to 3.46)
1	5 (14.7)	14 (46.7)	14 (40.0)		
2	8 (23.5)	7 (23.3)	7 (20.0)		
3	2 (5.9)	1 (3.3)	1 (2.9)		
4	1 (2.9)	0	1 (2.9)		
MR spectroscopy^c^					
Thalamic NAA concentration, mean (SD), mmol/kg wet weight^d^	10.98 (0.92)	8.36 (1.23)	9.02 (1.79)	−2.62 (−3.34 to −1.89)^e^	−1.96 (−2.66 to −1.26)^e^
NAA to choline peak area ratio, mean (SD)	1.70 (0.17)	2.02 (0.46)	1.71 (0.51)	0.32 (0.10 to 0.53)^e^	0.00 (−0.20 to 0.21)^e^
NAA to creatine peak area ratio, mean (SD)	0.89 (0.08)	0.88 (0.27)	0.97 (0.31)	−0.01 (−0.14 to 0.12)^e^	0.08 (−0.04 to 0.20)^e^
Thalamic lactate to NAA peak area ratio, median (IQR)	0.20 (0.17 to 0.24)	0.23 (0.16 to 0.33)	0.23 (0.19 to 0.31)	0.04 (−0.02 to 0.10)^f^	0.03 (−0.01 to 0.09)^f^

Abbreviations: MD, mean or median difference; MR, magnetic resonance; MRI, magnetic resonance imaging; NA, not applicable; NAA, *N*-acetyl aspartate; OR, odds ratio.

^a^ Data are presented as the number (percentage) of neonates unless otherwise indicated.

^b^ Odds ratios (calculated as the odds of being in the next-highest outcome category for the hypothermia group relative to the odds of being in the next-highest outcome category for the normothermia group) were reported for conventional MRI. Mean or median differences (calculated as cooling group values minus control group values) were reported for MR spectroscopy.

^c^ Normothermia, n = 31; 48-hour hypothermia, n = 26; and 72-hour hypothermia, n = 30.

^d^ NAA plus *N*-acetyl-aspartyl-glutamate methyl peaks were combined and referred to as NAA.

^e^ Mean difference.

^f^ Median difference.

Good-quality MR spectroscopy data for absolute quantification of NAA were available from 87 neonates (86.1%): 31 (91.1%), normothermia; 26 (83.9%), 48-hour hypothermia; and 30 (83.3%), 72-hour hypothermia. Clinical characteristics of neonates with vs without MR spectroscopy data are in eTable 2 in Supplement 2.

**Figure 2.**
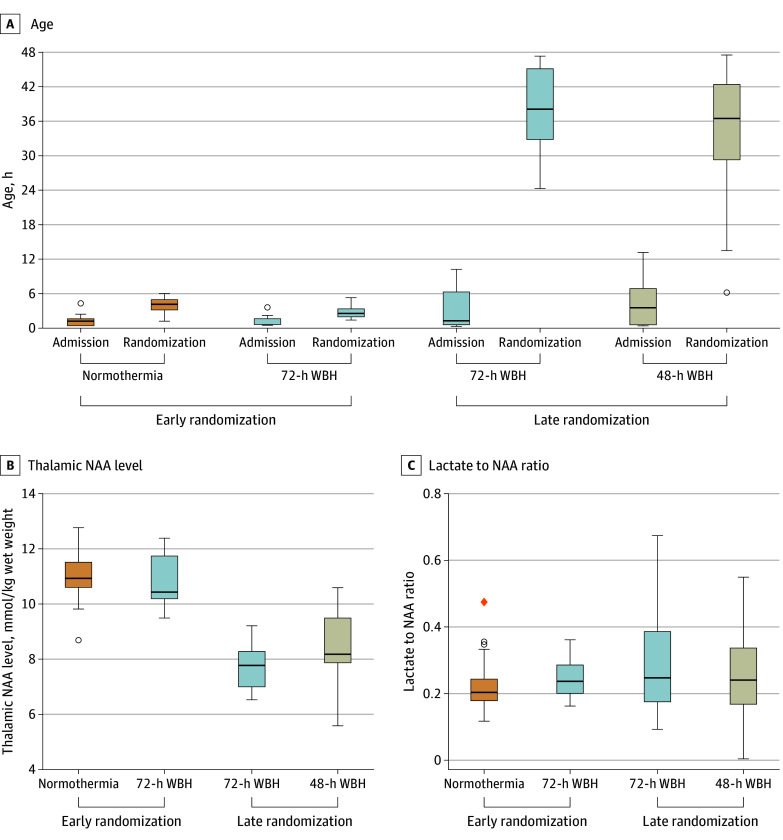
Age at Admission to the Cooling Center and Randomization, Thalamic *N*-Acetyl Aspartate (NAA) Levels, and Lactate to NAA Peak Area Metabolite Ratios Among Neonates Early indicates randomization within 6 hours; late, when the neonate was 6 hours or older. WBH indicates whole-body hypothermia. Medians are indicated by horizontal lines and the first and third quartiles by the lower and upper ends of the boxes. Whiskers indicate 1.5 times the IQRs from the first and third quartiles. Data more extreme than the whiskers are plotted individually as outliers (circles). One extreme outlier with a value more than 3 times the IQR above the third quartile is represented by an orange diamond.

Mean (SD) thalamic NAA concentrations in the 48-hour (8.36 [1.23] mmol/kg wet weight; mean difference, −2.62 [95% CI, −3.34 to −1.89] mmol/kg wet weight) and 72-hour (9.02 [1.79] mmol/kg wet weight; mean difference, −1.96 [95% CI, −2.66 to −1.26] mmol/kg wet weight) groups was lower than the normothermia group (10.98 [0.92] mmol/kg wet weight). Median (IQR) thalamic lactate to NAA metabolite peak area ratios in the 48-hour (0.23 [0.16-0.33]; median difference, 0.04 [95% CI, −0.02 to 0.10]) and 72-hour (0.23 [0.19-0.31]; median difference, 0.03 [95% CI, −0.01 to 0.09]) groups were not different from the normothermia group (0.20 [95% CI, 0.17-0.24]). Peak area ratios of other metabolites are in Table 3 and Figure 2.

Among the 48 neonates randomized within 6 hours, mean (SD) NAA level was 10.98 (0.92) mmol/kg wet weight in the normothermia and 10.77 (0.99) mmol/kg wet weight in the 72-hour hypothermia group (mean difference, −0.21 [95% CI, −0.88 to 0.46] mmol/kg wet weight). Among the 53 neonates randomized when 6 hours or older, mean (SD) NAA was 7.63 (0.85) mmol/kg wet weight in those who had 72 hours of hypothermia and 8.36 (1.23) mmol/kg wet weight in those rewarmed after 48 hours of hypothermia (mean difference, −0.69 [95% CI, −1.3 to −0.04] mmol/kg wet weight).

## Discussion

We report the feasibility and challenges of conducting a multicenter RCT of whole-body hypothermia for mild HIE using standardized neurological assessments and training. Brain injuries measured using cerebral MR biomarkers were not different in the 3 groups except for thalamic NAA, which was higher in the normothermic than in the 48- and 72-hour hypothermia groups. Occurrence of seizures 6 hours after birth was not different across groups. Use of invasive ventilation and opioids was higher in both hypothermia groups than the normothermia group. Although neonates in the hypothermia groups were sicker at baseline, these data suggest caution against offering whole-body hypothermia for neonates with mild HIE outside the context of an RCT.

To our knowledge, this is the first RCT of whole-body hypothermia for neonates with mild HIE. In a subgroup analysis of 47 neonates with mild HIE recruited to the observational Magnetic Resonance Biomarkers in Neonatal Encephalopathy (MARBLE) study,^25^ our group previously reported that thalamic NAA levels were not different in neonates with mild encephalopathy who had whole-body hypothermia for 72 hours compared with those with no hypothermia treatment. However, neonates who received whole-body hypothermia had reduced white matter injury on MRI (50% vs 87%; *P* = .02) and higher mean (SD) thalamic NAA to creatine peak area metabolite ratios (1.6 [0.21] vs 1.4 [0.1]; *P* < .001) than those who did not. At age 2 years, none of the whole-body hypothermia group had adverse neurodevelopmental outcomes compared with 2 noncooled neonates (14.3%) (*P* = .09).^25^ Pooled data from clinical trials of whole-body hypothermia that inadvertently recruited neonates with mild HIE (n = 117) did not show improvement in clinical outcomes at 2 years, although the CIs were wide to exclude significant harm or benefit.^26^

Despite lack of evidence on safety or efficacy, whole-body hypothermia is widely used for neonates with mild HIE in high-income countries. Data on 7181 neonates with mild HIE are available from the Canadian (n = 1089; cooled, 36%),^5^ California (n = 1364; cooled, 71%),^27^ Children’s Hospital Neonatal Consortium (2 reports: n = 945 [cooled, 13%]^28^ and n = 272 [cooled 95%]^29^), and UK (n = 3511; cooled, 30%)^30^ registries. In all of these registries, whole-body hypothermia (vs usual care) increased duration of ventilatory support (2 days vs 1 day), intensive care stay (9 vs 6 days), invasive ventilation (60% vs 45%), opioid infusion (67% vs 12%), disseminated intravascular coagulation (8% vs 2%), hepatic dysfunction (23% vs 11%), cardiac dysfunction (8% vs 2%), discharge home on oxygen (26% vs 15%), and tube feeding at hospital discharge (22% vs 13%). Other adverse short-term outcomes noted only in neonates with mild HIE who underwent whole-body hypothermia included hypotension (16%), thrombocytopenia (10%), coagulopathy (17%), persistent metabolic acidosis (8%), and subcutaneous fat necrosis (1%). No neurodevelopmental outcome data are available from these registries, so the long-term impact is unknown.

Given that almost 38% of neonates with mild HIE require special educational interventions at school age,^31,32,33^ evaluation of hypothermic neuroprotection is essential in this population. This pilot trial provides insights into the design of future clinical trials in the context of extensive therapeutic drift. Our data suggest that mild HIE can be identified within 6 hours of birth. However, this requires extensive training and certification in neurological assessment among frontline clinical staff at cooling centers and peripheral noncooling centers. As part of the trial, we developed a virtual training program and certification on modified Sarnat staging, which can be accessed through the National Health Service (NHS) learning system.^34^

Our data also highlight the spectrum of brain injury and disease severity within mild HIE; hence, nonrandomized designs will not be appropriate to evaluate neuroprotection. The better outcomes in the normothermic group in our trial may have resulted from optimal intensive care and monitoring, including continuous aEEG in a tertiary center and avoidance of hyperthermia. The latter is particularly important, as 14%^1,2^ to 29%^3^ of the original hypothermia trials reported hyperthermia in the control arms, thus overestimating hypothermic neuroprotection. Future RCTs should ensure optimal care for both hypothermic and normothermic arms. As most neonates with mild HIE are already being transferred to tertiary centers for hypothermia, additional burden on intensive care resources will likely be minimal.

A multicenter RCT (COMET) of 72-hour whole-body hypothermia (33.5 °C) or targeted normothermia (37 °C) involving 426 neonates with mild HIE from 40 NHS hospitals has been recently funded by the UK National Institute for Health and Care Research. The primary outcome is the cognitive composite scale score at 2 years of age. Once complete, the trial is expected to have 90% power to detect a clinically important difference between hypothermia and normothermia groups. The COMET investigators also plan to examine the impact of hypothermia on childhood outcome assessments, including special educational needs at school age. Another observational study (Comparative Effectiveness for Cooling Prospectively Infants With Mild Encephalopathy [COOLPRIME]) comparing outcomes of 430 neonates with mild HIE from 15 US hospitals has been funded by the Patient-Centered Outcomes Research Institute (PCORI).

### Limitations

This study has limitations. The pilot feasibility trial can only serve as a base for a larger RCT to evaluate safety and efficacy of whole-body hypothermia in reducing adverse outcomes at 2 years and beyond. The trial was neither powered nor intended to examine the safety and efficacy of hypothermia.

Second, although all neonates had mild encephalopathy and normal aEEG findings, baseline severity was higher among neonates randomized after vs before 6 hours of age. Hence, lower thalamic NAA levels may merely reflect differences in baseline disease severity.

Third, although prognostic accuracy of MR biomarkers and thalamic NAA for predicting adverse neurodevelopmental outcomes is established in neonates with moderate or severe HIE,^12^ the utility is not yet established in mild HIE. *N*-acetyl aspartate is a marker of neuronal integrity, and a reduction of 1 mmol/kg wet weight of thalamic NAA is associated with a reduction of 5 to 10 units in the Bayley cognitive composite score at 2 years in neonates with moderate or severe HIE.^12^ Hence, the reduction of almost 2 mmol/kg wet weight in thalamic NAA in the cooled neonates is of concern. Nevertheless, the mean thalamic NAA level in all 3 groups was well above the threshold of 5.6 mmol/kg wet weight for developing a moderate or severe disability. Clinical implications of NAA differences in normothermic and hypothermic groups are unclear and need to be correlated with cognitive outcomes at 2 years.

Fourth, we used thalamic NAA as an indirect marker of white matter injury rather than performing white matter spectroscopy due to challenges in absolute quantification of white matter NAA and constraints in scanning times. Fifth, 41.9% of neonates in the 48-hour and 58.3% in the 72-hour groups had shivering, and 83.9% in the 48-hour and 80.5% in the 72-hour groups received opioids vs none in the normothermia group. Preemptive opioid therapy during hypothermia increases ventilatory requirements and hospital stay and has not been shown to have any neuroprotective benefit.^35,36,37^

Sixth, recruitment and randomization occurred only at the time of admission to a tertiary NICU (cooling center). Only assessors based at these centers, not at the referring hospitals, were trained and certified in the neurological assessment.

## Conclusions

In this pilot RCT of neonates with mild HIE, whole-body hypothermia did not reduce brain injury as measured by quantitative MR biomarkers but increased need for invasive ventilation, hospital stay, and use of opioids. An RCT of whole-body hypothermia vs targeted normothermia is feasible in mild HIE despite extensive therapeutic drift but will require training and standardization of neurological assessment. Until safety and efficacy are established, we suggest that whole-body hypothermia should be offered to neonates with mild HIE only within the context of an RCT. Hypothermia should not be initiated without performing an adequate neurological assessment.

## Notes

Supplement 1.
Trial Protocol

Supplement 2. **eTable 1.** Baseline Characteristics in the Neonates Recruited Within 6 Hours After Birth and in Those Recruited At or After 6 Hours **eTable 2.** Baseline Characteristics of Neonates With Magnetic Resonance Spectroscopy Data and That Were Included in the Analysis, and Those Who Were Not **eFigure 1.** Recruitment Sites and Number of Neonates Randomized Within 6 Hours of Birth and At or After 6 Hours, Indicating Therapeutic Drift **eFigure 2.** Neurological Assessment at Recruitment Meeting the Eligibility Criteria for Mild Encephalopathy **eFigure 3.** Temperature Profile

Supplement 3.
COMET Trial Group

Supplement 4.
Data Sharing Statement
