# Supplementary material for: Whole-Body Hypothermia vs Targeted Normothermia for Neonates With Mild Encephalopathy: A Multicenter Pilot Randomized Clinical Trial
Source: JAMA Netw Open. 2024 May 6;7(5):e249119. doi: 10.1001/jamanetworkopen.2024.9119 (PMC11074808; doi:10.1001/jamanetworkopen.2024.9119)
Supplement: Supplement 1. — Trial Protocol [file jamanetwopen-e249119-s001.pdf]

Cooling in Mild Encephalopathy Trial (COMET)

Version 3
Date: 27/10/2021
IRAS ID: 241031
JRCO Reference: 18HH4385

Protocol authorised by:
Name & Role: Dr Sudhin Thayyil, Chief Investigator (Reader and Head of Academic Neonatology, Imperial College London)

Date: 27/10/2021

Signature:

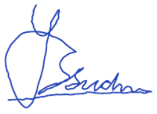

This protocol describes the COMET trial and provides information about procedures for entering participants. Every care was taken in its drafting, but corrections or amendments may be necessary. These will be circulated to investigators in the study. Problems relating to this study should be referred, in the first instance, to the Chief Investigator. This study will adhere to the principles outlined in the NHS Research Governance Framework for Health and Social Care (2nd edition). It will be conducted in compliance with the protocol, the Data Protection Act and other regulatory requirements as appropriate.

Chief Investigator:
Sudhin Thayyil MD, DCH, FRCPCH, PhD
Reader and Hon Consultant Neonatologist
Head of Academic Neonatology
Director, Centre for Perinatal Neuroscience
Department of Paediatrics
Imperial College London and Imperial Neonatal Service
Du Cane Road | London W12 0HS | (44) 203 313 8515
https://www.imperial.ac.uk/perinatal-neuroscience/

For all queries, please contact:
Josephine Mendoza; R514, 5th Floor Hammersmith House, Hammersmith Hospital
Du Cane Rd, London W12 0HS; Tel: 0044 20 3313 2473 - Email: j.mendoza@imperial.ac.uk

Sponsor:
Imperial College London is the Sponsor for this study. For further information regarding the sponsorship conditions, please contact the Head of Regulatory Compliance at:

Joint Research Compliance Office
Imperial College London and Imperial College Healthcare NHS Trust
Room 215, Level 2, Medical School Building
Norfolk Place
London, W2 1PG
Tel 0207 594 1872

Funder:
National Institute for Health Research and Garfield Weston Foundation, Imperial College London

INDEX

1. STUDY SUMMARY 4
3. AIMS 8
4. PATIENTS AND METHODS 9
  4.1 Study participants 9
  4.2 Inclusion criteria 9
  4.3 Study procedures 10
  4.4 Withdrawal criteria 12
  4.5. Diagnosis of neonatal seizures 12
  4.6 Outcome measures 12
5. ADVERSE EVENTS 12
6. ASSESSMENT AND FOLLOW-UP 14
8. REGULATORY ISSUES 14
  8.1 Regulatory & ethics approval 14
  8.2 Consent 14
  8.3 Confidentiality 14
  8.4 Indemnity 15
  8.5 Sponsor 15
  8.6 Funding 15
  8.7 Audits 15
9. STUDY MANAGEMENT 15
10. Protocol Compliance and Breaches of GCP 15
11. Publications policy 16

1. STUDY SUMMARY

Although therapeutic hypothermia for 72 hours reduces brain injury and improves long term neurodevelopmental outcomes after moderate or severe neonatal encephalopathy[1-3], the benefits and optimal duration[4] of cooling therapy in mild encephalopathy is not known[5]. Adverse neurodevelopmental outcomes at 2 years occur in 16% of babies with un-treated mild neonatal encephalopathy. In the phase I of the COMET trial, we have shown that it is feasible to identify and randomise babies with mild encephalopathy, and to obtain the primary outcome (proton MR spectroscopy levels of Thalamic N-acetyl Aspartate) accurately. The phase II of the COMET trial will examine the benefits and optimal duration of cooling therapy in babies with mild encephalopathy.

Currently, most centres in the UK routinely cool babies with mild encephalopathy, either knowingly or inadvertently[6]. The former is primarily driven by a fear of legal implications of missing the therapeutic window, in case a baby with mild encephalopathy develops seizures after six of age and progress to moderate encephalopathy, while the latter is driven by a lack of awareness of the original cooling trial criteria and expertise in structured neurological examination in neonatal encephalopathy[7]. A small minority of the UK centres accurately perform a neurological examination and aEEG within six hours of birth, and do not offer cooling therapy to babies with normal aEEG. Hence, the COMET trial will be conducted in two different cohorts, so that the key questions can be addressed in the most effective way with minimal disruption to the existing clinical practices of the recruiting sites.

Research questions

1. Does whole body cooling initiated within 6 hours of birth and continued for 72 hours increase thalamic MR spectroscopy N-acetyl aspartate levels in babies with mild encephalopathy, when compared with those who are not cooled? (Cohort 1)
2. In babies with mild encephalopathy undergoing cooling therapy as clinical care, does rewarming at 48 hours as opposed to 72 hours result in similar thalamic N-acetyl aspartate levels? (Cohort 2)

Study Population

Cohort 1: A total of 60 babies with mild encephalopathy ($\geq$36 weeks; $\geq$2Kg) aged less than 6 hours will be recruited from several tertiary neonatal units in the UK, Europe, USA and Canada, over a 2 year period. The babies will be randomised to usual care (no cooling) or cooling therapy (core temperature 33 to 34 C) for 72 hours within six hours of birth. MR imaging and spectroscopy will be performed between 4 to 14 days after birth.

Cohort 2: A total of 80 babies will mild encephalopathy ($\geq$36 weeks; $\geq$2Kg) aged 24 to 48 hours and undergoing cooling therapy as a part of standard clinical care will be recruited from several UK cooling centres, over a 2 year period. The babies will be randomised to rewarming after 48 hours or 72 hours of cooling therapy. MR imaging and spectroscopy will be performed between 4 to 14 days after birth. The babies recruited to cohort 1 will not be eligible for recruitment to cohort 2.

Primary outcome (both cohorts)

- Proton MR spectroscopy Thalamic N-acetyl aspartate levels between 4 to 14 days of age.

Benefits of the trial

These data will inform the national and international guidelines on management of babies with mild neonatal encephalopathy. If a shorter duration of cooling is as good or better than 3 days of cooling, this will reduce the intensive care stays, opioid use[8] and separation from parents.

2. BACKGROUND

Birth asphyxia related brain injury (neonatal encephalopathy, NE) occurs in 4 to 6 per 1000 live births, and is the most common cause of death and neuro-disability in term babies in the UK [9,10]. Moderate whole-body cooling, to a core temperature of 33.5°C for 72 hours, reduces death and disability after moderate or severe NE, and is used as the standard of care treatment for these babies across the NHS, and in other high-income countries. As these trials primarily focussed on babies with moderate or severe encephalopathy, the safety and efficacy of cooling therapy in babies with mild encephalopathy remains unknown. Two recent meta-analysis of cooling trials in babies with mild encephalopathy did not show any change in the clinical outcomes (death or moderate or severe disability) at 18 months, although the total number of babies included were too small to exclude a therapeutic effect.

Brain injury and outcomes after mild encephalopathy

50% of affected babies with mild encephalopathy have brain injury on magnetic resonance (MR) imaging [4,11-13], and one third are reported to have an adverse long term neurodevelopmental outcome. Jacobs et al. (2011) reported adverse neurodevelopmental outcomes at two years of age in 8/24 (33%) children who had mild NE at birth and were not cooled [14]. Murray et al. (2016) reported significantly lower full scale IQ, verbal IQ and performance IQ in 22 children following mild NE, when compared with 30 healthy controls, assessed at five years of age ($p<0.005$) [15]. Lally et al. (2014) have reported neurodevelopmental delays at 3.5 years of age in 8/24 (33%) south Indian babies with mild NE [16]. Such neurodevelopmental impairments will have a profound lifelong impact, including an increased risk of autism [17], attention deficit disorders [18] and learning difficulties [19-21].

The most accurate data on later outcomes after un-treated mild encephalopathy comes from the multicentre PRIME (Prospective research in mild encephalopathy) study. The PRIME study reported adverse neurodevelopmental outcomes at 2 years in 16% of babies with un-treated mild neonatal encephalopathy. Of these approximately 40% had severe adverse outcomes (Bayley III cognitive composite score <70; Gross motor function classification (GMFCS): 3 to 5) and 60% had mild adverse outcomes (Bayley III cognitive 70–84 or ≥85 and either GMFCS 1 or 2, seizures, or hearing deficit)[22,23].

Current practice of cooling therapy for mild encephalopathy

In the UK, a web based survey of 54 of the 68 UK cooling centres, reported that 36 (67%) routinely cooled babies with mild NE babies (who by definition do not meet the original cooling criteria)[24]. Although all centres used the same depth of cooling (33 to 34°C), the duration of cooling therapy varied widely – 39% discontinued cooling by 24h if there was a clinical improvement, and 61% cooled for the full 72h irrespective of clinical improvement [25]. Separately, a recent audit of the London Neonatal Transport Service (NTS) reported a similar therapeutic drift, as 41% (59/145) of babies transported for cooling therapy in London had only mild or no NE [26]. The situation in the USA is very similar. A survey in 2017, reported that all except 2 centres routinely offered cooling to babies with mild encephalopathy.

There are two key reasons for this therapeutic creep. Firstly, the NICHD neonatal research network cooling trial reported in 2005, used a standardised neurological examination without aEEG as the inclusion criteria for cooling. This criterion is extensively validated and accurately identifies babies with moderate and severe encephalopathy who are risk of adverse outcome, however it does require training and certification in the neurological examination.

Unfortunately, the TOBY trial published 4 years later, did not use a structured neurological examination, and instead mandated an abnormal aEEG for cooling. Hence, in the UK, most cooling centres never developed expertise in performing a structured NICHD neurological examination. When

cooling became the standard of care in the UK, most UK centres omitted aEEG as a cooling criterion and started offering cooling therapy based on an abnormal neurological examination alone. Without adequate training or certification in the NICHD neurological examination, most clinicians misclassify babies with mild encephalopathy as moderate and inadvertently cool babies these infants, despite most of them having a normal aEEG. In addition, many babies with perinatal asphyxia are transferred from peripheral hospitals to the cooling centres without an adequate neurological examination prior to initiation of cooling therapy. The clinicians at the cooling centres are then faced with a dilemma of continuing the therapy for full three days or discontinuing the therapy when the clinical picture is consistent with mild encephalopathy. Even when aEEG is used, clinicians are often unaware of the remarkably low specificity (less than 50%) of an early aEEG, and often mistake a moderate voltage abnormality in aEEG with moderate encephalopathy. Published data suggest that moderate or severe aEEG abnormalities can occur even in babies with mild encephalopathy, although it is unlikely that a baby with normal aEEG will have moderate of severe encephalopathy. Thus, clinicians offering cooling therapy to babies with normal aEEG are in fact cooling babies without encephalopathy or with mild encephalopathy.

Secondly, even if a structured neurological examination within six hours of birth is consistent with mild encephalopathy and aEEG is normal, a small proportion (approximately 5 to 10%) of these babies may develop seizures after six hours of age and progress to moderate or severe encephalopathy. This is because the brain injury following perinatal asphyxia evolves over the first 24 hours, and at present there are no effective ways of predicting which babies will worsen and develop seizures after six hours. Therefore, not initiating cooling therapy within six hours may be seen as a missed opportunity. Clinicians who are concerned about legal implications if these infants develop adverse outcomes later, tend to practice defensive medicine and offer cooling to any baby with abnormal neurological examination after perinatal asphyxia.

Preclinical data and the optimal duration of cooling therapy for mild encephalopathy
Preclinical models have consistently shown effective neuroprotection with cooling after moderate or severe NE [27], and more recently after mild NE [28]. The brain injury evolves slowly after mild NE [29], and hypothermia is remarkably neuroprotective in such cases [30], when compared with severe brain injury [27]. Hence it is possible that a shorter duration of cooling therapy would be sufficient to provide neuroprotection in mild NE.

However, the minimal duration of cooling for neuroprotection is species-dependent. Hence the optimal durations of therapeutic hypothermia in animal models and humans are different. For example, small animal models of NE (mice, rats) require shorter cooling durations and large animal models (pig, sheep) require longer cooling durations. Hence, most preclinical studies have observed neuroprotection with 24h to 48 h of cooling, while very short periods or very long periods of cooling are not neuroprotective [31].

Considering the rapid clinical recovery that occurs in mild NE, and the potential for over-ventilation and hypocarbia which could worsen brain injury, it is likely that these babies may benefit from a duration of cooling that is shorter than the full 72 hours. Such an approach would also reduce the length of intensive care stays and the need for prolonged sedation. Nevertheless, cooling for less than 24 hours is unlikely to be beneficial, and we have found MRI evidence of residual brain injury in 50% of the babies with mild encephalopathy who were cooled for less than 24, and long term adverse outcomes in 20% [4].

Safety and efficacy of cooling therapy in mild encephalopathy: meta-analysis.
We conducted a systematic review and meta-analysis of all cooling trials involving babies with mild neonatal encephalopathy (Figure 1). The pooled data including 117 babies with mild encephalopathy

showed an odd ratio of 1.12 (95% CI 0.42 to 2.98), and hence we need further evidence before recommending cooling as a standard therapy for these babies. Adverse neurodevelopmental outcomes at 18 months were seen in 20% of the babies with mild encephalopathy.

Figure 1. The effect of cooling on adverse outcomes (death, moderate or severe disability) after mild neonatal encephalopathy[5]

	Cooling		Usual care			Odds Ratio	
**Study or Subgroup**	**Events**	**Total**	**Events**	**Total**	**Weight**	**M-H, Random, 95% CI**	**Year**
Battin 2001 (SHC)	1	5	0	6	8.2%	4.33 [0.14, 132.32]	2001
Gluckman 2005 (SHC)	1	5	1	3	9.1%	0.50 [0.02, 12.90]	2007
Zhou 2010 (SHC)	1	21	1	18	11.8%	0.85 [0.05, 14.64]	2010
Jacobs 2011 (WBC)	4	16	8	24	47.8%	0.67 [0.16, 2.74]	2011
Thayyil 2013 (WBC)	4	9	2	10	23.2%	3.20 [0.42, 24.42]	2013
**Total (95% CI)**		**56**		**61**	**100.0%**	**1.12 [0.42, 2.98]**	
Total events	11		12				

Heterogeneity: $Tau^2 = 0.00$; $Chi^2 = 2.42$, df = 4 (P = 0.66); $I^2 = 0\%$
Test for overall effect: Z = 0.23 (P = 0.82)

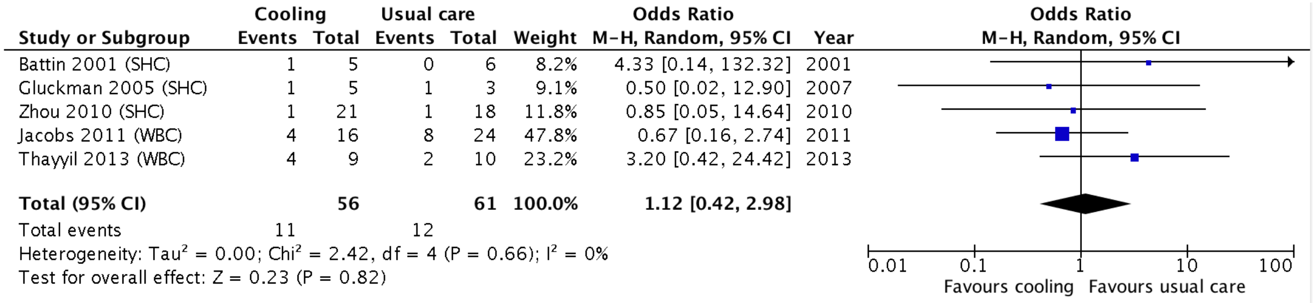

Surrogate MR biomarkers of long-term outcome in neonatal encephalopathy

An extensive systematic review and meta-analysis of the published literature suggested that MR spectroscopy biomarkers, measured shortly after birth, have the highest accuracy in predicting adverse outcomes years after neonatal encephalopathy [32]. However, the published studies were based on single centre studies which are of limited use for multi-centre trials.

As a part of the MARBLE study we developed cross-platform MR spectroscopy techniques and prospectively examined the prognostic accuracy of various MR biomarkers in a multi-country setting. We found that the absolute concentration of thalamic N-acetylaspartate, [NAA], measured within two weeks of birth, had a near perfect 97% specificity and 100% sensitivity in predicting an adverse neurological outcome at 2 years after neonatal encephalopathy[33]. Thalamic [NAA] at two weeks accounted for 40% of the variance observed in cognitive performance scores at two years of age, and significantly out-performed metabolite peak area ratios such as lactate/NAA, and other MR biomarkers including fractional anisotropy measurements.

Brain injury after cooling therapy in babies with mild neonatal encephalopathy

We examined the brain injury in 41 babies with mild neonatal encephalopathy recruited into the MARBLE study. Of these, 27 babies were cooled for 72 hours (‘full’) and 14 were not cooled (‘none’) or were cooled for less than 6 hours (‘partial’). Although the cooled babies were sicker, they had less white matter injury on conventional MR imaging and higher NAA/choline levels on MR spectroscopy[34].

Figure 2. Thalamic MR spectroscopy metabolites (left) and Bayley III composite scores (right) of cooled and non-cooled babies with mild encephalopathy.

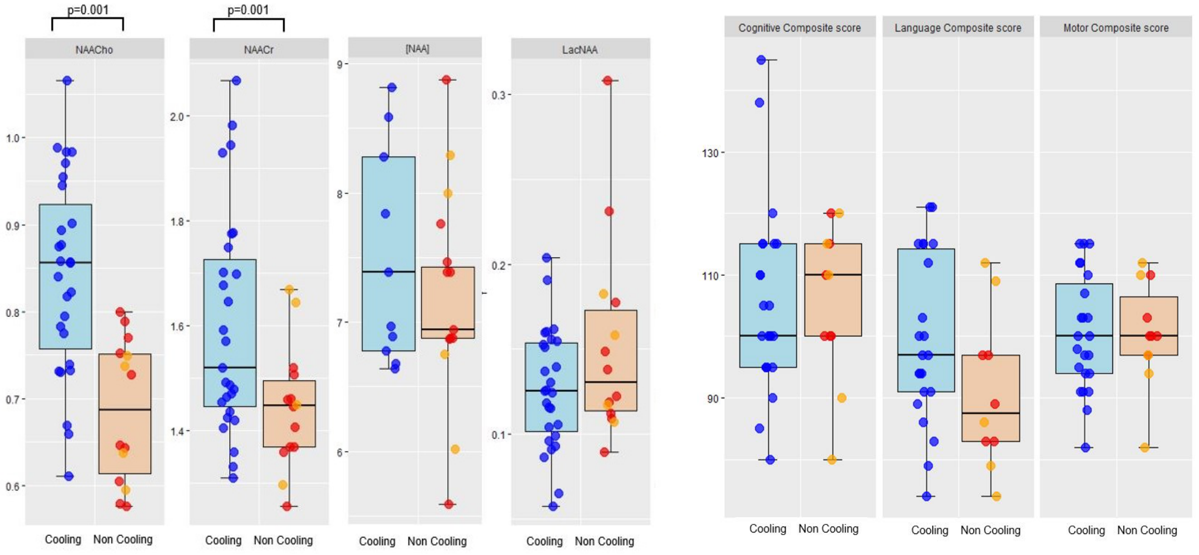

Justification of the COMET trial design

Given that the duration of cooling is species-dependent, it is unlikely that the optimal duration of cooling in animal models can be directly extrapolated to humans. The conventional approach of evaluating neuroprotective interventions in phase III trials is challenging in mild encephalopathy due to several reasons. For example, approximately 760 babies with mild encephalopathy need to be recruited per arm to detect a 30% reduction in the adverse outcomes (moderate or severe disability or death) at 80% power and 0.05% significance, given a control event rate of 20%. This requires a massive international effort and resources, and treatment failures at phase III trials are catastrophic. An alternative approach is to examine the mean differences in cognitive scores as an outcome measure. Such trials would still require large sample sizes and may not provide adequate information for health policy changes and the NICE guidelines.

In the COMET trial, we will use thalamic [NAA] as a surrogate biomarker to identify the benefits and optimal duration of cooling therapy in mild encephalopathy.

3. AIMS

Primary aims

1. To examine if whole body cooling initiated within 6 hours of birth and continued for 72 hours increase thalamic MR spectroscopy N-acetyl aspartate levels in babies with mild encephalopathy, when compared with those who are not cooled (Cohort 1)
2. To examine if, in babies with mild encephalopathy undergoing cooling therapy as clinical care, rewarming at 48 hours as opposed to 72 hours result in similar thalamic N-acetyl aspartate levels (Cohort 2)

Secondary aims

1. To compare the brain injury on conventional MR imaging and peak area metabolite ratios of NAA/Creatine and NAA/Choline in cooled and non-cooled babies with mild encephalopathy.
2. To compare the brain injury on conventional MR imaging and peak area metabolite ratios of NAA/Creatine and NAA/Choline with 48 hours and 72 hours of cooling therapy in mild encephalopathy.
3. To compare the mean duration of hospital stay following no cooling, 48 hours and 72 hours of cooling therapy

4. To compare the host gene expression changes following no cooling, 48 hours and 72 hours of cooling therapy

4. PATIENTS AND METHODS

All tertiary neonatal centres providing therapeutic hypothermia and with facilities for 3 Tesla or 1.5 Tesla MRI scanning and spectroscopy are eligible to participate in the COMET trial.

4.1 Study participants

Babies (>35 weeks gestation and birth weight >1.8kg) requiring resuscitation at birth will be screened for eligibility and recruited if they meet the inclusion criteria. We will recruit a total of 140 term or near term (>35 weeks) babies (Cohort 1: 60 babies; Cohort 2: 80 babies) with mild neonatal encephalopathy over a 24 month period.

4.2 Inclusion criteria

All of the following three criteria should be met:

<u>Cohort 1:</u> A total of 60 babies with mild encephalopathy (≥36 weeks; ≥2Kg) aged less than 6 hours will be recruited from several tertiary neonatal units in the UK, Europe, USA and Canada, over a 2 year period. The babies will be randomised to usual care (no cooling) or cooling therapy (core temperature 33 to 34 C) for 72 hours within six hours of birth. MR imaging and spectroscopy will be performed between 4 to 14 days after birth.

<u>Inclusion criteria</u>

All of the following three criteria should be met:

1. Age less than six hours.

   AND

2. Evidence of acute perinatal asphyxia
   a. Metabolic acidosis (pH <7.0 and/or BE >-16) in cord gas or a blood gas within one hour of birth.

      OR

   b. If the pH or BE is borderline (pH<7.15 to 7.0) and/or BE >-10 to -16) in cord and/or blood gas within 1h of birth, or no blood gas available, additional evidence of perinatal asphyxia is required, which includes either an acute obstetric event (e.g. cord prolapse, abruption, shoulder dystocia) OR Need for continued resuscitation or ventilation at 10 minutes and/or a 10 min Apgar score <6

   AND

3. Evidence of mild encephalopathy (atleast two abnormalities) on the structured NICHD neurological examination performed between 1 and 6h of birth.

<u>Exclusion criteria</u>

1. Babies without encephalopathy.
2. Babies with moderate or severe encephalopathy who meets the original NIHCD[2] or the TOBY[1] trial cooling trial criteria.
3. Babies with definite or probable seizures as per the recent ILAE classification[35].
4. Babies with moderate or severe abnormalities on aEEG voltage criteria.
5. Babies with life threatening congenital malformations.

<u>Cohort 2:</u> A total of 80 babies will mild encephalopathy (≥36 weeks; ≥2Kg) aged 24 to 48 hours and undergoing cooling therapy as a part of clinical care of the recruiting centre will be recruited from several UK cooling centres, over a 2 year period. If the baby is eligible, parents will be approached for

recruitment between 24 to 48 hours after birth. The site investigator will review the baby's progress and aEEG over the first 24 hours, perform an NICHD neurological exam and will randomise the baby to rewarming after 48 hours or 72 hours of cooling therapy, if the baby meets the eligibility criteria. The chief investigator at Imperial College London will review the clinical and aEEG data if there is a dilemma about the trial eligibility.

Once the baby is recruited, MR imaging and spectroscopy will be performed between 4 to 14 days after birth, as in cohort 1. The babies recruited to cohort 1 will not be eligible for recruitment to cohort 2.

<u>Inclusion criteria</u>

1. Cooling therapy initiated within six hours of birth and continued beyond 24 hours by the clinical team on a baby who do not meet the original NICHD neonatal research network[2] or the TOBY Trial[1] Cooling criteria.

2. Evidence of acute perinatal asphyxia
    a. Metabolic acidosis (pH <7.0 and/or BE >-16) in cord gas or a blood gas within one hour of birth.

       OR

    b. If the pH or BE is borderline (pH<7.15 to 7.0) and/or BE >-10 to -16) in cord and/or blood gas within 1h of birth, or no blood gas available, additional evidence of perinatal asphyxia is required, which includes either an acute obstetric event (e.g. cord prolapse, abruption, shoulder dystocia) OR Need for continued resuscitation or ventilation at 10 minutes and/or a 10 min Apgar score <6.

<u>Exclusion criteria</u>

- Babies with moderate or severe encephalopathy who meets the original NIHCD[2] or the TOBY trial[8] cooling trial criteria.
- Babies with definite or probable seizures as per the recent ILAE classification at any stage until randomisation[35].
- Babies with moderate or severe abnormalities on aEEG voltage criteria at any stage until randomisation.
- Babies with life threatening congenital malformations

4.3 Study procedures

Cohort 1: After informed parental consent a total of 60 babies with mild encephalopathy aged less than six hours will be randomised to usual care or whole-body cooling (rectal temperature 33.5°C $\pm$ 0.5°C) for 72 hours. The babies in the usual care arm will have skin temperature monitoring instead of rectal temperature.

Cohort 2: The babies who are being cooled for encephalopathy without meeting the original cooling criteria (i.e. having a normal aEEG and/or a clinical examination consistent with mild encephalopathy) will be recruited between 24 to 48 hours of age. The babies will be randomised to rewarming at 48 hours or at 72 hours, at a rate of 0.5 C per hour.

Randomisation (web-based) will be performed at the cooling centre for all babies. The standard clinical referral pathway will be followed for out-born babies with mild NE, and the clinical assessment and randomisation will happen only at the cooling centre. However, the transport teams will provide the information leaflets and discuss the study with the parents prior to retrieval, so that the babies can be randomised immediately on admission.

Supportive treatment will be the same for all groups, as per the unit protocol. All babies will have amplitude integrated EEG within six hours of birth, for a minimum period of 24 hours, as part of standard clinical care. Following completion of cooling therapy, babies in the cooling arm will be rewarmed at a rate of 0.5°C per hour to eventually reach normothermia.

<u>Use of sedation during cooling</u>
Although sedation is often used during cooling in the UK, there is little evidence based to support this. In a non-randomised study, Thoresen et al. (2001) compared the brain injury in 18 piglets kept normothermic (39°C) with 21 piglets who were cooled to 35°C for 24 hours [36]. The piglets were not given general anaesthesia, and no difference in brain injury was seen. The cooled piglets shivered and had elevated cortisol levels. The lack of neuroprotection was attributed to stress, possibly due to inadequate sedation, although the study examined anaesthetics rather than sedatives. Unlike piglets, newborn babies have brown fat and hence non-shivering thermogenesis occurs in response to cooling. Thus, the clinical implications of these data are unclear [36].

Furthermore, neuroprotection is observed in other animal models without sedation [37]. More recent data from the sub-analysis of the NICHD cooling trial suggest that sedation had no effect on the neurodevelopmental outcomes [38]. Secondary analysis of the MARBLE study shows routine pre-emptive opioid sedation prolongs hospital stay and increases hypotension, without any beneficial effects on neuroprotection or later neurodevelopmental outcomes. Hence, routine sedation will not be mandated in the COMET trial, and babies would be extubated whenever clinically stable.

<u>Babies developing seizures after randomisation</u>
A small number of babies (less than 5% in the PRIME study and MARBLE study) with mild encephalopathy during the initial assessment, may develop seizures and progress to moderate encephalopathy.

Recent evidence from delayed cooling trial suggests possibility of some neuroprotection even up to 24 hours of age[39]. Hence, any baby developing seizures or progressing to moderate encephalopathy will receive cooling therapy for at least 72 hours as per the unit protocol. The data from these babies will be analysed as per the original allocation (Intention to treat).

<u>aEEG and ECG</u>
All babies will have aEEG as a part of the routine clinical care for the first 24 hours. In addition, 2 channel ECG data from the first 24 hours will be stored on a small portable device for analysis of heart rate variability.

<u>Blood samples</u>
We will collect 1 ml of blood at the time of randomisation and again at 80 hours from randomisation from all recruited babies. The blood (venous or arterial) will be collected, at the time of routine clinical sampling, whenever possible.

<u>MR imaging and spectroscopy</u>
All recruited babies will have 3 Tesla MR imaging and spectroscopy acquired between 4 and 7 days of age (prior to hospital discharge whenever possible). In addition to conventional T_1 and T_2, and diffusion tensor imaging, a series of spectroscopy data will be acquired in a 15x15x15mm^3 voxel centred on the thalamus to quantify NAA concentration[33]. Due to the variance arising from inter-site scanner differences, an MR spectroscopy phantom will be used for calibration across all sites prior to starting the study. In the absence of a comprehensive diffusion phantom, inter-site diffusion data will be corrected post-hoc using ComBat [40]. The results of the MR scans including any incidental findings will be fed back to the clinical teams for appropriate counselling of the parents.

4.4 Withdrawal criteria

Withdrawal should occur only upon parental or clinician request. Recent data from the NICHD delayed cooling trial suggests potential benefits of cooling therapy for up to 24 hours of age in babies with moderate or severe encephalopathy. Hence, any baby who develops documented seizures (clinical and/or aEEG) between the age of 6 to 24 hours (i.e. after randomisation) will be considered to have moderate or severe encephalopathy, and will receive full cooling therapy for at least 72 hours, at the discretion of attending clinical teams [39]. These babies will not be excluded from the study, and will be analysed in the originally allocated group (intention to treat).

4.5. Diagnosis of neonatal seizures

Clinical diagnosis can miss almost half of the seizures in neonatal encephalopathy. Conversely, normal mannerisms may be misclassified as seizures if EEG is not used. Hence, the recent classification of neonatal seizures by the International league against epilepsy (ILAE) mandates the use of EEG or aEEG in neonatal intensive care units[35]. Neonatal seizures are classified based on 5 levels of diagnostic certainty.

> Level 1 (Definite seizures) – Seizures confirmed on conventional EEG with (electro-clinical seizure) or without (electro-graphic only) clinical manifestations.
>
> Level 2a (Probable seizures) – Seizures confirmed on aEEG with (electro-clinical seizure) or without clinical manifestations (electro-graphic only)
>
> Level 2b (Probable seizures) – Clinically assessed focal clonic or focal tonic seizure directly witnessed or reviewed on video by experienced medical personnel when EEG or aEEG was not available.
>
> Level 3: (Possible seizure) – Clinical events suggestive of epileptic seizures other than focal clonic or focal tonic seizures, directly witnessed or reviewed on video by experienced medical personnel
>
> Leve 4: (Not seizure) – Reported seizure event (as previously defined) but insufficient evidence to meet the case definition
>
> Level 5: (Not seizure) – Reported seizure event (as previously defined), documented or witnessed by experienced medical personnel and evaluated by simultaneous conventional EEG or aEEG and determined NOT to be a case of neonatal seizure.

4.6 Outcome measures

- Mean thalamic [NAA] levels.
- Mean thalamic NAA/Choline and Creatine peak area metabolite ratios.
- Brain injury score on conventional MR imaging.
- Mean hospital stay.

5. ADVERSE EVENTS

All known adverse events relating to neonatal encephalopathy and whole-body cooling are described in the parent information leaflet and will be discussed at the point of obtaining the informed research consent, prior to the start of cooling. The following clinical events occur due to the underlying disease (neonatal encephalopathy) although these are less likely in babies with mild neonatal encephalopathy. Previous data from cooling trials on babies with moderate and severe encephalopathy suggest that these adverse outcomes are reduced by cooling.

1. Brain injury on magnetic resonance imaging
2. Death during the neonatal period or during infancy
3. Adverse neurodevelopmental outcome at 18 months and at childhood
4. Persistent pulmonary hypertension
5. Metabolic imbalances

6. Cardiac arrhythmia
7. Thrombocytopenia
8. Renal failure
9. Coagulopathy

Whole-body cooling may increase the risk of the following adverse events.
1. Thrombocytopenia and an increased need for platelet transfusions
2. Subcutaneous fat necrosis

All adverse events are expected to occur within the cooling period (first 72 hours) or within 72 hours of re-warming. Adverse reactions occurring subsequently (after 1 week of life), except subcutaneous fat necrosis, will not be considered as relating to the intervention. Subcutaneous fat necrosis may occur several weeks after the intervention.

Serious adverse events that may be due to hypothermia are:
- Cardiac arrhythmia.
- Life-threatening bleeds
- Major venous thrombosis not related to an infusion line

6.1 DEFINITIONS

Adverse Event (AE): any untoward medical occurrence in a patient or clinical study subject.
Serious Adverse Event (SAE): any untoward and unexpected medical occurrence or effect that:
- Results in death
- Is life-threatening – refers to an event in which the subject was at risk of death at the time of the event; it does not refer to an event which hypothetically might have caused death if it were more severe
- Requires hospitalisation, or prolongation of a current inpatient's hospitalisation
- Results in persistent or significant disability or incapacity
- Is a congenital anomaly or birth defect

Medical judgement should be exercised in deciding whether an AE is serious in other situations, or performed by a delegated person according to the study delegation log. Important AEs that are not immediately life-threatening or do not result in death or hospitalisation (but may jeopardise the subject or require intervention to prevent one of the other outcomes listed in the definition above), should also be considered serious.

6.2 REPORTING PROCEDURES

All adverse events will be recorded during hospitalisation using the case report form. Babies who suffer neonatal encephalopathy are expected to have higher mortality and morbidity up to two years of age. If an UNEXPECTED serious adverse event occurs (i.e. an event not mentioned in the above list in 6.), it should be reported to the COMET trial manager at the Centre for Perinatal Neuroscience (CPN) at Imperial College London within 24 hours, using one of the Serious Adverse Event report forms. The COMET trial manager will ensure that the COMET Independent Data Monitoring Committee and the Research Ethics Committee are informed accordingly.

The Chief Investigator must notify the Sponsor of all unexpected SAEs. If there is any unexpected SAE which would be considered study related, the Chief Investigator will report to the Ethics Committee within 15 days of becoming aware of the event, using the SAE form. Local investigators should report any SAEs as required by their Sponsor and/or Research & Development Office.

6. ASSESSMENT AND FOLLOW-UP

7.1 Neurodevelopmental evaluation

All babies will have detailed neurodevelopmental evaluation including Bayley Scales of Infant Development (BSID-III or IV if available), Gross Motor Function Classification System (GMFCS), and hearing and vision assessment between 18 to 24 months of age. Severe disability will be defined as any one of the following: both cognitive and language composite BSID-III scores <70, GMFCS level 3–5, hearing impairment requiring hearing aids, or blindness. Moderate disability will be defined as both cognitive and language composite BSID-III scores between 70 and 84 and one or more of the following: GMFCS level 2, hearing impairment with no need for amplification or a persistent seizure disorder. The outcomes will also be assessed by categorisation of these scores and by including mortality as an outcome. An adverse outcome will be defined as death or, in survivors, moderate or severe disability.

We will also send parents an adapted Parent Report of Children's Abilities – Revised (PARCA- R) at 2 years of age. It is a parent completed questionnaire that can be used to assess children's cognitive and language development. This tool will be sent to parents from both cohorts (HIE and Healthy) to obtain reference values for the questionnaire. This is important to ensure the study gathers long term follow-up data for all participants, regardless of clinical practice as well as capturing developmental difficulties that may be perceived by parents but not detected in BSID-III[41]. PARCA has been validated to measure cognitive abilities and behavioural adjustment and is available non commercially[42].

8. REGULATORY ISSUES

8.1 Regulatory & ethics approval

This study has been reviewed and approved by the Health Research Authority and the West of Scotland Research Ethics Committee. The study will be conducted in accordance with the recommendations for physicians involved in research on human subjects adopted by the 18th World Medical Assembly, Helsinki 1964 and later revisions. The study will also receive confirmation of capacity and capability from each participating NHS Trust before accepting participants into the study.

8.2 Consent

The clinical team (including the neonatal transport team) will explain the study to the parents and will provide the parent information leaflet in the first instance. Informed parental consent (in person or over telephone) will be obtained prior to recruitment and randomisation.

Whether or not the parent(s) decide to take part in the study shall not affect the clinical decisions made during the care of the baby, nor the quality of care provided. All participants are free to withdraw from the study at any time without giving any reason and without prejudicing further treatment.

8.3 Confidentiality

The Chief Investigator will preserve the confidentiality of participants taking part in the study according to the Data Protection Act, UK. Personal identification data including telephone numbers and all contact details will be stored: (i) as hardcopies in a research folder in locked cupboards in the site Principal Investigator's office, and the Imperial College London research office; (ii) on NHS computers at the recruiting sites (only for babies recruited from that site); and (iii) on a secure and encrypted server at Imperial College London.

All personal data will be stored for a period of 10 years, and will be destroyed using standard Imperial College London protocols (including removal by specialist software for electronic data), unless parental consent for further research is obtained at that time.

All MR data will be stripped of identifying information upon export from the scanner, and all other records only reference study numbers. Any remaining potentially sensitive data which are necessary to know for the purposes of study (e.g. date of birth) will be kept on a central, backed-up and encrypted drive on the Imperial College London network, which is only accessible from a separate physical location. Access to each of these areas is tightly controlled, and new users requiring access to these data will require formal authorisation from the Chief Investigator.

MR data will be again anonymised using the study number, and encrypted with a password prior to transfer. Imperial College London file transfer protocols will be used for data transfer. All research data will be stored at Imperial College London, for a period of 10 years.

8.4 Indemnity

Imperial College London holds insurance policies for both negligent harm and non-negligent harm, which apply to this study.

8.5 Sponsor

Imperial College London will act as the main Sponsor for this study. Delegated responsibilities will be assigned to the NHS trusts taking part in this study.

8.6 Funding

The National Institute for Health Research, UK and Garfield Weston Foundation funds this study. There are no payments offered to the study participants.

8.7 Audits

The study may be subject to inspection and audit by Imperial College London under their remit as sponsor and other regulatory bodies to ensure adherence to both GCP and the NHS Research Governance Framework for Health and Social Care (2nd edition).

9. STUDY MANAGEMENT

The day-to-day management of the study will be co-ordinated through the Centre for Perinatal Neuroscience, Imperial College London. The Project Management Group (PMG) will oversee all aspects of the day-to-day running of the study, and will consist of the investigators and the COMET trial staff, based at the Centre for Perinatal Neuroscience, Imperial College London. PMG will hold a monthly teleconference of all COMET investigators for the entire duration of the trial to discuss the data quality and recruitment.

The responsibilities of the PMG include:

- Appointment and training of the local research staff for the COMET trial
- Case recruitment at participating centres
- Distribution and supply of data collection forms and other appropriate documentation for the trial
- Data collection and management
- Organisation of the follow-up
- Data entry and cleaning
- Collection of adverse event data

10. Protocol Compliance and Breaches of GCP

Prospective, planned deviations or waivers to the protocol are not allowed under the UK regulations on clinical trials and must not be used. For example, it is not acceptable to enrol a subject if they do not meet one or more eligibility criteria or restrictions specified in the trial protocol.

Protocol deviations, non-compliances, or breaches are departures from the approved protocol. They can happen at any time, but are not planned. They must be adequately documented on the relevant forms and reported to the Chief Investigator and Sponsor immediately. Deviations from the protocol which are found to occur constantly again and again will not be accepted and will require immediate action and could potentially be classified as a serious breach. Any potential/suspected serious breaches of GCP must be reported immediately to the Sponsor without any delay.

11. Publications policy

Ownership of the data arising from this trial resides with the trial team. On completion of the trial the data will be analysed and tabulated and a final study report prepared. Consort guidelines and checklists are reviewed prior to generating any publications for the trial to ensure they meet the standards required for submission to high quality peer reviewed journals etc. http://www.consort-statement.org/

A copy of the study results will be also given to the parents of all recruited babies, if they wish. This will be recorded at the time of recruitment, and again during follow up. The study sponsor and funders will have no role in the study management, analysis and interpretation of data, writing of the report or the decision to submit the report for publication.

Appendix 1. NICHD Examination for neonatal encephalopathy

Eligibility criteria require ≥ 2 abnormal categories in mild and NOT ≥3 moderate or severe.

CATEGORIES	NORMAL	MILD	MODERATE	SEVERE
**1. Level of consciousness**	Alert, responsive to external stimuli	Hyper-alert, has a stare, jitteriness, high pitched cry, exaggerated response to minimal stimuli, inconsolable	Lethargic	Stupor, Coma
**2. Spontaneous activity**	Normal	Decreased, with or without periods of excessive activity	Decreased	No activity
**3. Posture**	Predominantly flexed when quiet	Mild flexion of distal joints (fingers, wrist)	Strong distal flexion, complete extension	Intermittent decerebration
**4. Tone**	Strong flexor tone in all extremities	Slightly increased peripheral tone	Hypotonia or Hypertonia	Flaccid or Rigid
**5. Reflexes**				
**Suck**	Strong, easy to elicit	Weak, Poor	Weak or has bite	Absent
**Moro**	Strong, easy to elicit	Low threshold to elicit	Incomplete	Absent
**6. Autonomic Nervous System**				
**Pupils**	Normal size	Mydriasis	Miosis	Deviation/Dilated/ Non-reactive
**Heart rate**	Normal heart rate	Tachycardia (>160)	Bradycardia (<100/minute)	Variable heart rate
**Respirations**	Normal	Hyperventilation (>80/min)	Periodic breathing	Apnea or on ventilator with or without spontaneous respirations

REFERENCES

1. Azzopardi DV, Strohm B, Edwards AD, et al. Moderate hypothermia to treat perinatal asphyxial encephalopathy. *N Engl J Med* 2009; **361**(14): 1349-58.

2. Shankaran S, Laptook AR, Ehrenkranz RA, et al. Whole-body hypothermia for neonates with hypoxic-ischemic encephalopathy. *N Engl J Med* 2005; **353**: 1574-84.

3. Jacobs SE, Berg M, Hunt R, Tarnow-Mordi WO, Inder TE, Davis PG. Cooling for newborns with hypoxic ischaemic encephalopathy. *Cochrane Database Syst Rev* 2013; **1**: CD003311.

4. Lally PJ, Montaldo P, Oliveira V, et al. Residual brain injury after early discontinuation of cooling therapy in mild neonatal encephalopathy. *Arch Dis Child Fetal Neonatal Ed* 2018; **103**(4): F383-F7.

5. Kariholu U, Montaldo P, Markati T, et al. Therapeutic hypothermia for mild neonatal encephalopathy: a systematic review and meta-analysis. *Arch Dis Child Fetal Neonatal Ed* 2018.

6. Oliveira V, Singhvi DP, Montaldo P, et al. Therapeutic hypothermia in mild neonatal encephalopathy: a national survey of practice in the UK. *Arch Dis Child Fetal Neonatal Ed* 2018; **103**(4): F388-F90.

7. Natarajan G, Laptook A, Shankaran S. Therapeutic Hypothermia: How Can We Optimize This Therapy to Further Improve Outcomes? *Clinics in perinatology* 2018; **45**: 241-55.

8. Liow N, Montaldo P, Lally PJ, et al. Preemptive Morphine During Therapeutic Hypothermia After Neonatal Encephalopathy: A Secondary Analysis. *Ther Hypothermia Temp Manag* 2019.

9. Gale C, Statnikov Y, Jawad S, Uthaya SN, Modi N, Brain Injuries expert working g. Neonatal brain injuries in England: population-based incidence derived from routinely recorded clinical data held in the National Neonatal Research Database. *Arch Dis Child Fetal Neonatal Ed* 2017.

10. Excellence NIfHaC. Therapeutic hypothermia with intracorporeal temperature monitoring for hypoxic perinatal brain injury: guidance *NICE guidelines: interventional procedures* 2010; **http://www.nice.org.uk/nicemedia/live/11315/48809/48809.pdf**(IPG 347): ISBN 978-1-84936-251-1.

11. Walsh BH, Neil J, Morey J, et al. The Frequency and Severity of Magnetic Resonance Imaging Abnormalities in Infants with Mild Neonatal Encephalopathy. *J Pediatr* 2017.

12. DuPont TL, Chalak LF, Morriss MC, Burchfield PJ, Christie L, Sanchez PJ. Short-term outcomes of newborns with perinatal acidemia who are not eligible for systemic hypothermia therapy. *J Pediatr* 2013; **162**(1): 35-41.

13. Gagne-Loranger M, Sheppard M, Ali N, Saint-Martin C, Wintermark P. Newborns Referred for Therapeutic Hypothermia: Association between Initial Degree of Encephalopathy and Severity of Brain Injury (What About the Newborns with Mild Encephalopathy on Admission?). *Am J Perinatol* 2016; **33**(2): 195-202.

14. Jacobs SE, Morley CJ, Inder TE, et al. Whole-body hypothermia for term and near-term newborns with hypoxic-ischemic encephalopathy: a randomized controlled trial. *Arch Pediatr Adolesc Med* 2011; **165**(8): 692-700.

15. Murray DM, O'Connor CM, Ryan CA, Korotchikova I, Boylan GB. Early EEG Grade and Outcome at 5 Years After Mild Neonatal Hypoxic Ischemic Encephalopathy. *Pediatrics* 2016; **138**(4).

16. Lally PJ, Price DL, Pauliah SS, et al. Neonatal encephalopathic cerebral injury in South India assessed by perinatal magnetic resonance biomarkers and early childhood neurodevelopmental outcome. *PLoS One* 2014; **9**(2): e87874.

17. Getahun D, Fassett MJ, Peltier MR, et al. Association of Perinatal Risk Factors with Autism Spectrum Disorder. *American journal of perinatology* 2017; **34**(3): 295-304.

18. Getahun D, Rhoads GG, Demissie K, et al. In utero exposure to ischemic-hypoxic conditions and attention-deficit/hyperactivity disorder. *Pediatrics* 2013; **131**(1): e53-61.

19. de Haan M, Wyatt JS, Roth S, Vargha-Khadem F, Gadian D, Mishkin M. Brain and cognitive-behavioural development after asphyxia at term birth. *Dev Sci* 2006; **9**(4): 350-8.

20. van Handel M, de Sonneville L, de Vries LS, Jongmans MJ, Swaab H. Specific memory impairment following neonatal encephalopathy in term-born children. *Dev Neuropsychol* 2012; **37**(1): 30-50.

21. van Handel M, Swaab H, de Vries LS, Jongmans MJ. Behavioral outcome in children with a history of neonatal encephalopathy following perinatal asphyxia. *J Pediatr Psychol* 2010; **35**(3): 286-95.

22. Prempunpong C, Chalak LF, Garfinkle J, et al. Prospective research on infants with mild encephalopathy: the PRIME study. *J Perinatol* 2018; **38**(1): 80-5.

23. Chalak LF, Nguyen KA, Prempunpong C, et al. Prospective research in infants with mild encephalopathy identified in the first six hours of life: neurodevelopmental outcomes at 18-22 months. *Pediatr Res* 2018; **84**(6): 861-8.

24. Oliveira V, Singhvi DP, Montaldo P, et al. Therapeutic hypothermia in mild neonatal encephalopathy: a national survey of practice in the UK. *Arch Dis Child Fetal Neonatal Ed* 2017.

25. Oliveira V, Singhvi D, Montaldo P, et al. Therapeutic hypothermia in mild neonatal encephalopathy: a national survey of practice in the U.K. . Neonatal Society Meeting. Brighton, UK; 2017.

26. Goel N, Mohinuddin S, Ratnavel N, Sinha A. Neurological assessment in infants referred for therapeutic hypothermia: a need for more structured assessment and /or pragmatic criteria? European Congress of Perinatal Medicine https://london-ntsnhsuk/wp-content/uploads/2015/03/Neurological-Assesment-ECPM-Poster-659-2014pdf; 2014; Florence; 2014.

27. Wood T, Osredkar D, Puchades M, et al. Treatment temperature and insult severity influence the neuroprotective effects of therapeutic hypothermia. *Sci Rep* 2016; **6**: 23430.

28. Koo E, Sheldon RA, Lee BS, Vexler ZS, Ferriero DM. Effects of therapeutic hypothermia on white matter injury from murine neonatal hypoxia-ischemia. *Pediatr Res* 2017.

29. Beilharz EJ, Williams CE, Dragunow M, Sirimanne ES, Gluckman PD. Mechanisms of delayed cell death following hypoxic-ischemic injury in the immature rat: evidence for apoptosis during selective neuronal loss. *Brain Res Mol Brain Res* 1995; **29**(1): 1-14.

30. Koo E, Sheldon RA, Lee BS, Vexler ZS, Ferriero DM. Effects of therapeutic hypothermia on white matter injury from murine neonatal hypoxia-ischemia. *Pediatr Res* 2017; **82**(3): 518-26.

31. Davidson JO, Draghi V, Whitham S, et al. How long is sufficient for optimal neuroprotection with cerebral cooling after ischemia in fetal sheep? *J Cereb Blood Flow Metab* 2017: 271678X17707671.

32. Thayyil S, Chandrasekaran M, Taylor A, et al. Cerebral magnetic resonance biomarkers in neonatal encephalopathy: a meta-analysis. *Pediatrics* 2010; **125**(2): e382-95.

33. Lally PJ, Montaldo P, Oliveira V, et al. Magnetic resonance spectroscopy assessment of brain injury after moderate hypothermia in neonatal encephalopathy: a prospective multicentre cohort study. *Lancet neurology* 2019; **18**(1): 35-45.

34. Montaldo P, Lally PJ, Oliveira V, et al. Therapeutic hypothermia initiated within 6 hours of birth is associated with reduced brain injury on MR biomarkers in mild hypoxic-ischaemic encephalopathy: a non-randomised cohort study. *Arch Dis Child Fetal Neonatal Ed* 2018.

35. Pressler R, Cilio MR, Mizrahi E, et al. The ILAE Classification of Seizures & the Epilepsies: Modification for Seizures in the Neonate. Proposal from

the ILAE Task Force on Neonatal Seizures. *Vaccine* (In press): https://www.ilae.org/files/dmfile/NeonatalSeizureClassification-ProofForWeb.pdf.

36. Thoresen M, Satas S, Løberg E, et al. Twenty-four hours of mild hypothermia in unsedated newborn pigs starting after a severe global hypoxic-ischemic insult is not neuroprotective. *Pediatr Res* 2001; **50**(3): 405-11.

37. Wassink G, Lear CA, Gunn KC, Dean JM, Bennet L, Gunn AJ. Analgesics, sedatives, anticonvulsant drugs, and the cooled brain. *Semin Fetal Neonatal Med* 2015; **20**(2): 109-14.

38. Natarajan G, S S, A L, et al. Association between Sedation-Analgesia and Neurodevelopment Outcomes in Neonatal Hypoxic-ischemic Encephalopathy. *Journal of Perinatology* 2018; **(in press)**.

39. Laptook AR, Shankaran S, Tyson JE, et al. Effect of Therapeutic Hypothermia Initiated After 6 Hours of Age on Death or Disability Among Newborns With Hypoxic-Ischemic Encephalopathy: A Randomized Clinical Trial. *JAMA* 2017; **318**(16): 1550-60.

40. Fortin JP, Parker D, Tunc B, et al. Harmonization of multi-site diffusion tensor imaging data. *Neuroimage* 2017; **161**: 149-70.

41. Martin AJ, Darlow BA, Salt A, Hague W, Sebastian L, McNeill N, et al. Performance of the Parent Report of Children's Abilities-Revised (PARCA-R) versus the Bayley Scales of Infant Development III. Arch Dis Child. 2013;98(12):955-8.

42. Johnson S, Bountziouka V, Brocklehurst P, Linsell L, Marlow N, Wolke D, et al. Standardisation of the Parent Report of Children's Abilities-Revised (PARCA-R): a norm-referenced assessment of cognitive and language development at age 2 years. Lancet Child Adolesc Health. 2019;3(10):705-12.
