# Supplementary material for: Whole-Body Hypothermia vs Targeted Normothermia for Neonates With Mild Encephalopathy: A Multicenter Pilot Randomized Clinical Trial
Source: JAMA Netw Open. 2024 May 6;7(5):e249119. doi: 10.1001/jamanetworkopen.2024.9119 (PMC11074808; doi:10.1001/jamanetworkopen.2024.9119)
Supplement: Supplement 2. — eTable 1. Baseline Characteristics in the Neonates Recruited Within 6 Hours After Birth and in Those Recruited At or After 6 Hours eTable 2. Baseline Characteristics of Neonates With Magnetic Resonance Spectroscopy Data and That Were Included in the Analysis, and Those Who Were Not eFigure 1. Recruitment Sites and Number of Neonates Randomized Within 6 Hours of Birth and At or After 6 Hours, Indicating Therapeutic Drift eFigure 2. Neurological Assessment at Recruitment Meeting the Eligibility Criteria for Mild Encephalopathy eFigure 3. Temperature Profile [file jamanetwopen-e249119-s002.pdf]

Supplementary Online Content

Montaldo P, Cirillo M, Burgod C, et al; COMET Trial Group. Whole-body hypothermia vs targeted normothermia for neonates with mild encephalopathy: a multicenter pilot randomized clinical trial. *JAMA Netw Open*. 2024;7(5):e249119. doi:10.1001/jamanetworkopen.2024.9119

eTable 1. Baseline Characteristics in the Neonates Recruited Within 6 Hours After Birth and in Those Recruited At or After 6 Hours

eTable 2. Baseline Characteristics of Neonates With Magnetic Resonance Spectroscopy Data and That Were Included in the Analysis, and Those Who Were Not

eFigure 1. Recruitment Sites and Number of Neonates Randomized Within 6 hours of Birth and At or After 6 Hours, Indicating Therapeutic Drift

eFigure 2. Neurological Assessment at Recruitment Meeting the Eligibility Criteria for Mild Encephalopathy

eFigure 3. Temperature Profile

This supplementary material has been provided by the authors to give readers additional information about their work.

© 2024 Montaldo P et al. *JAMA Network Open*.

eTable 1. **Baseline characteristics in the neonates recruited within 6 hours after birth and in those recruited at or after 6 hours.**

	**Neonates randomised within 6 hours of birth**		**Neonates randomised at or after 6 hours of birth**	
**Characteristic**	**Normothermia (n=34), No. (%)**	**Hypothermia 72 hours (n=14), No. (%)**	**Hypothermia 48 hours (n=31), No. (%)**	**Hypothermia 72 hours (n=22), No. (%)**
**Antenatal history**				
Mean (SD) maternal age (years)	30.9 (5.9)	30.6 (7.1)	32.1 (5.3)	31.8 (6.1)
Primigravida	16 (47)	7 (50.0)	18 (58)	8 (36.3)
Reduced fetal movements	1/25 (4.0)	0/11 (0.0)	3/24 (12.5)	1/16 (6.2)
CTG abnormalities	16/21 (76.1)	8/11 (72.7)	10/28 (35.7)	8/19 (42.1)
Meconium staining	15/34 (44.1)	4 (28.5)	8 (25.8)	4/20 (20.0)
Maternal pyrexia	4/33 (12.1)	1 (7.1)	4/30 (13.3)	1 (5.5)
Prolonged rupture of membranes	2/34 (5.8)	2 (14.2)	2 (6.4)	1 (4.5)
**Perinatal sentinel events** *(events were not mutually exclusive)*	22/33(66.6)	11 (78.5)	16/30 (53.3)	11 (50.0)
Cord mishap	14 (42)	6 (42.8)	3 (10.0)	3 (13.6)
Lengthened second stage	3 (9.0)	1 (7.1)	2 (6.6)	0 (0.0)
Obstructed labor	2 (6.0)	2 (14.2)	4 (13.3)	3 (13.6)
Shoulder dystocia	4 (11.8)	1 (7.1)	3 (9.6)	1 (4.5)
Antepartum hemorrhage	1 (2.9)	0 (0.0)	3(10.0)	4 (18.1)
Uterine rupture	0 (0.0)	0 (0.0)	1 (3.3)	2 (9.0)
**Delivery**				
Instrumental delivery	6 (17.6)	3 (21.4)	6 (19.3)	5 (22.7)
Emergency caesarean	9 (26.4)	5 (35.7)	13 (41.9)	11 (50.0)
**Birth**				
Inborn	28 (82.3)	13 (92.8)	13 (41.9)	10 (45.4)
Male	19 (55.9)	6 (42.8)	18 (58.1)	11 (50.0)
Mean (SD) gestation, weeks	39.5 (1.1)	39.1 (0.8)	38.7 (0.5)	39.2 (1.4)
Mean (SD) birthweight, g	3378 (380)	3347 (244)	3017 (338)	3191 (469)
Mean (SD) cord arterial blood pH	7.0 (0.1)	7.0 (0.06)	7.0 (0.2)	7.0 (0.1)
Median (IQR) Apgar score at 5 min	7.0 (6.0-8.0)	6.0 (6.0-8.0)	5 (3.0-7.0)	5.0 (3.0-8.0)
Median (IQR) Apgar score at 10 min	8.0 (7.0-9.0)	8.0 (8.0-9.0)	7.0 (6.0-9.0)	7.0 (5.0-8.0)
**Delivery room resuscitation**				
Intubation	3 (8.8)	3 (21.4)	14 (45.1)	10 (45.4)
Epinephrine	0 (0.0)	0 (0.0)	1 (3.2)	0 (0.0)
Chest compression	0 (0.0)	0 (0.0)	5 (16)	0 (0.0)
Extubation within 3 hours	1 (2.9)	0 (0.0)	5 (16)	2 (9.0)
**Neonatal course and adverse events**				
Invasive ventilation	3 (8.8)	4 (28.5)	14 (45.1)	12 (54.5)
Median (IQR), invasive ventilation duration, hours	3 (2-3)	25 (14-38)	7 (1-16)	20 (9-41)
Non-invasive ventilation[a]	8 (23.5)	2 (14.2)	1 (3.2)	3 (13.6)
Median (IQR), non-invasive ventilation duration, hours[a]	3 (2-3)	66.5 (56-78)	18 (7-29)	19 (11-37)
Opioid use	0 (0.0)	12 (85.7)	26 (83.8)	17 (77.3)
Shivering	0 (0.0)	7 (50)	13 (41.9)	14 (63.6)
Hypotension requiring inotropes	1 (2.9)	2 (14.2)	0 (0.0)	2 (9.1)
Persistent metabolic acidosis	0 (0.0)	1 (7.1)	0 (0.0)	1 (4.5)

© 2024 Montaldo P et al. *JAMA Network Open*.

Subcutaneous fat necrosis	0 (0.0)	0 (0.0)	1 (3.2)	0 (0.0)
Thrombocytopenia requiring platelets	1 (2.9)	1 (7.1)	1 (3.2)	1 (4.5)
Abnormal clotting	1 (2.9)	3 (21.4)	0 (0.0)	1 (4.5)
Blood stream infection	1 (2.9)	1 (7.1)	1 (3.2)	0 (0.0)
Seizures after 6 hours of age	1 (2.9)	1 (7.1)	1 (3.2)	1 (4.5)
Median (IQR), postnatal age at MRI (days) [c]	5.8 (5-7)	6.8 (5.7-8.0)	4 (3.5-5.8)	6.9 (4.8-9.5)
Median (IQR), hospital stay (days)	5.9 (3.7-6.6)	8.7 (7.2-9.1)	6.2 (4.8-7.9)	8.9 (5.8-10.5)
Death	0 (0.0)	1 (7.1)	0 (0.0)	0 (0.0)
**MR spectroscopy**				
Thalamic NAA, mean (SD), mmol/kg wet weight	10.97 (0.92)	10.77 (0.99)	8.27 (1.22)	7.63 (0.85)
NAA to choline peak area ratios, mean (SD)	1.70 (0.16)	1.72 (0.18)	1.99 (0.44)	1.75 (0.66)
NAA to creatinine peak area ratios, mean (SD)	0.89 (0.08)	0.89 (0.08)	0.88 (0.26)	0.99 (0.38)
Thalamic lactate to NAA peak area ratios, mean (SD)	0.21 (0.07)	0.24 (0.05)	0.24 (0.11)	0.28 (0.14)

[a] Non-invasive ventilation was defined as the need for continuous positive airway pressure or high flow oxygen

© 2024 Montaldo P et al. *JAMA Network Open*.

eTable 2. Baseline characteristics of neonates with magnetic resonance spectroscopy (MRS) data and that were included in the analysis, and those who were not.

**Characteristic**	**Babies with MRS data (n=87), No. (%)**	**Babies without MRS data (n=14), No. (%)**
**Antenatal history**		
Mean (SD) maternal age (years)	31.2 (6)	32.9 (6)
Primigravida	39 (44.8)	10 (71.4)
Reduced fetal movements	3 (3.4)	2 (14.2)
CTG abnormalities	34 (39.0)	8 (57.1)
Meconium staining	25 (28.7)	6 (42.8)
Maternal pyrexia	7 (8.0)	3 (21.4)
Prolonged rupture of membranes	5 (5.7)	2 (14.2)
**Perinatal sentinel events** *(events were not mutually exclusive)*	52 (59.7)	8 (57.1)
Cord mishap	24 (27.5)	2 (14.2)
Lengthened second stage	5 (5.7)	1 (7.1)
Obstructed labor	10 (11.4)	1 (7.1)
Shoulder dystocia	8 (9.1)	1 (7.1)
Antepartum hemorrhage	5 (5.7)	3 (21.4)
Uterine rupture	2 (2.2)	1 (7.1)
**Delivery**		
Instrumental delivery	18 (20.7)	2 (14.3)
Emergency caesarean	27 (31.0)	11 (78.6)
**Birth**		
Inborn	52 (59.7)	12 (85.7)
Male	46 (52.8)	8 (57.1)
Mean (SD) gestation, weeks	39.3 (1.2)	39.0 (1.5)
Mean (SD) birthweight, g	3355 (393)	3159 (441)
Mean (SD) cord arterial blood pH	7.0 (0.1)	6.9 (0.1)
Median (IQR) Apgar score at 5 min	6 (5.0-8.0)	4.0 (3.0-8.0)
Median (IQR) Apgar score at 10 min	8 (7.0-9.0)	7.0 (6.0-9.0)
**Delivery room resuscitation**		
Intubation	21 (24.1)	9 (64.2)
Epinephrine	0 (0.0)	0 (0.0)
Chest compression	0 (0.0)	0 (0.0)

© 2024 Montaldo P et al. *JAMA Network Open*.

eFigure 1. Recruitment sites and number of neonates randomised within 6 hours of birth (red) and at or after 6 hours (blue) indicating therapeutic drift

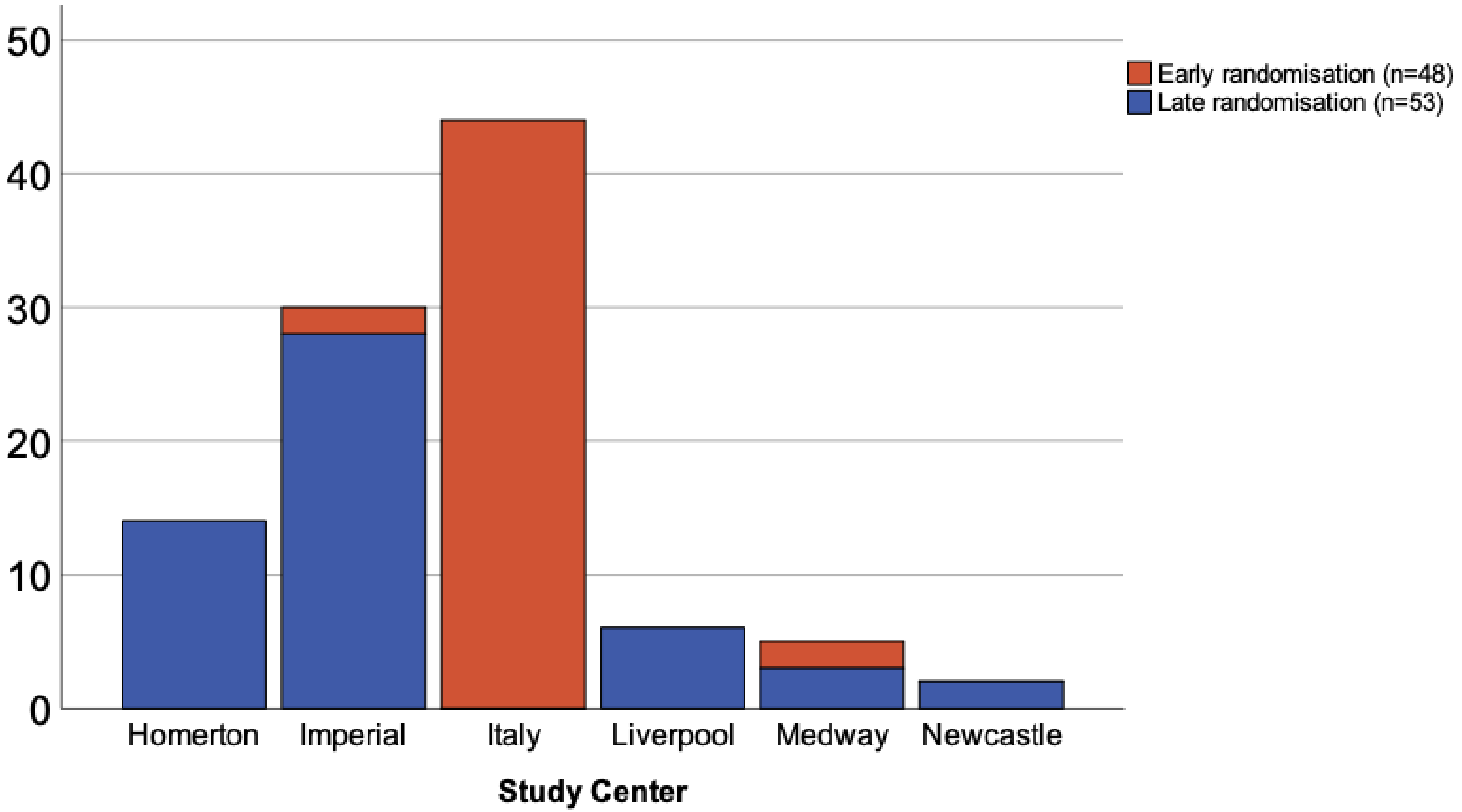

© 2024 Montaldo P et al. *JAMA Network Open*.

eFigure 2. Neurological assessment at recruitment meeting the eligibility criteria for mild encephalopathy.

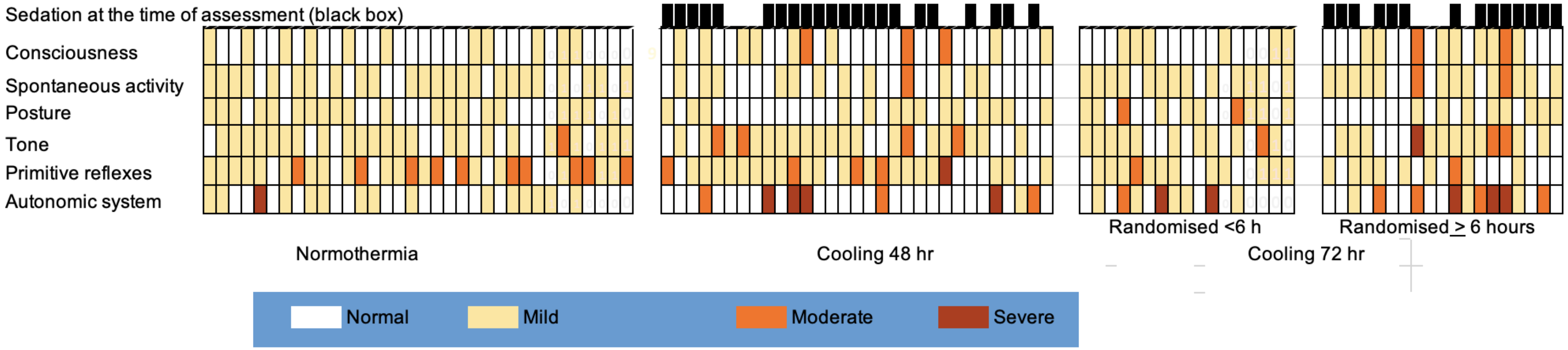

© 2024 Montaldo P et al. *JAMA Network Open*.

eFigure 3. Temperature profile. Mean (SD) rectal temperature of the neonates randomized to the 48-hour and the 72-hour and hypothermia groups and skin temperature in the normothermia group.

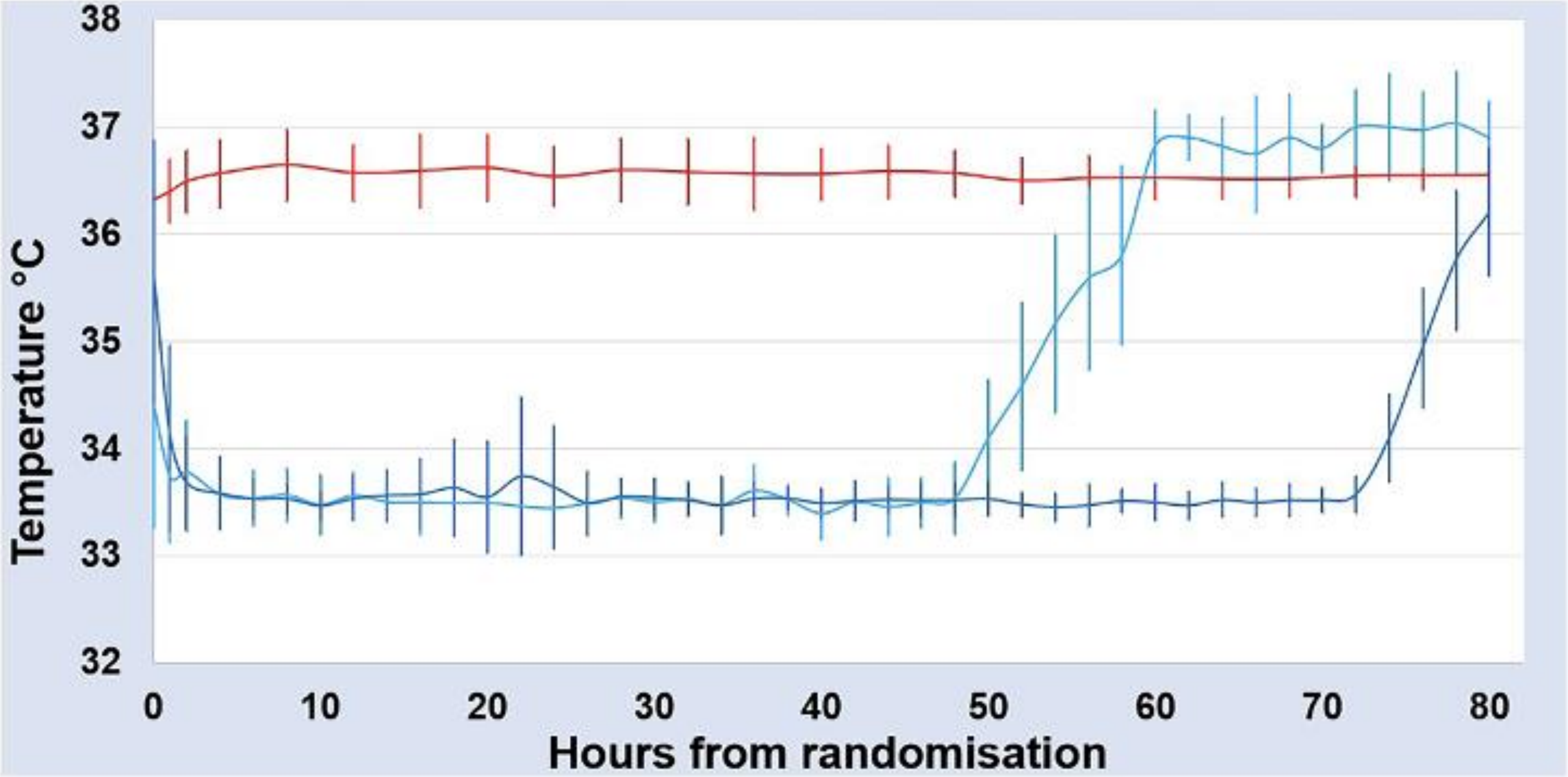

© 2024 Montaldo P et al. *JAMA Network Open*.
