# Supplementary material for: Whole-Body Hypothermia vs Targeted Normothermia for Neonates With Mild Encephalopathy: A Multicenter Pilot Randomized Clinical Trial
Source: JAMA Netw Open. 2024 May 6;7(5):e249119. doi: 10.1001/jamanetworkopen.2024.9119 (PMC11074808; doi:10.1001/jamanetworkopen.2024.9119)
Supplement: Supplement 3. — COMET Trial Group [file jamanetwopen-e249119-s003.pdf]

Supplemental Online Content: Nonauthor Collaborators
*First name, last name, and suffix (if applicable) are required and will appear in PubMed.

*Group Name(s): COMET Trial Group

***First Name and Middle Initial(s)**	***Last Name**	***Suffix (eg, Jr, III)**	Academic Degrees	Institution	Location (city, state/province, country)	Role or Contribution, eg, chair, principal investigator	Group (if more than 1 Group listed in the byline) and/or Subgroup (eg, Steering Committee)
Emanuele	Miraglia del Giudice	NA	MD, PhD	University of Campania Luigi Vanvitelli	Naples	Chair	
Carlo	Capristo	NA	MD, PhD	University of Campania Luigi Vanvitelli	Naples	Principal investigator	
Margherita	Internicola	NA	MD	University of Campania Luigi Vanvitelli	Naples	Recruitment	
Anna	Maietta	NA	MD	University of Campania Luigi Vanvitelli	Naples	Recruitment	
Giuseppe	Montefusco	NA	MD	University of Campania Luigi Vanvitelli	Naples	Recruitment	
Umberto	Pugliese	NA	MD	University of Campania Luigi Vanvitelli	Naples	Recruitment	
Ferdinando	Spagnuolo	NA	MD	University of Campania Luigi Vanvitelli	Naples	Recruitment	
Mario	Diplomatico	NA	MD	AORN San Giuseppe Moscati	Avellino	Recruitment	
Sabino	Moschella	NA	MD	AORN San Giuseppe Moscati	Avellino	Principal investigator	
