# Supplementary material for: Whole-Body Hypothermia vs Targeted Normothermia for Neonates With Mild Encephalopathy: A Multicenter Pilot Randomized Clinical Trial
Source: JAMA Netw Open. 2024 May 6;7(5):e249119. doi: 10.1001/jamanetworkopen.2024.9119 (PMC11074808; doi:10.1001/jamanetworkopen.2024.9119)
Supplement: Supplement 4. — Data Sharing Statement [file jamanetwopen-e249119-s004.pdf]

Data Sharing Statement

Montaldo. Whole-Body Hypothermia vs Targeted Normothermia for Neonates With Mild Encephalopathy. *JAMA Netw Open*. Published May 06, 2024.
doi:10.1001/jamanetworkopen.2024.9119

Data
Data available: No

Additional Information
Explanation for why data not available: Additional secondary papers are currently being written up. Once these are reported the data will be made publicly available.
